# Towards automatic freeform optics design: coarse and fine search of the three-mirror solution space

**DOI:** 10.1038/s41377-021-00510-z

**Published:** 2021-03-29

**Authors:** Benqi Zhang, Guofan Jin, Jun Zhu

**Affiliations:** State Key Laboratory of Precision Measurement Technology and Instruments, Department of Precision Instrument, Tsinghua University, Beijing, 100084 China

**Keywords:** Applied optics, Imaging and sensing

## Abstract

Design of an optical system, whether classic or novel, in the past or the present, requires significant effort from the designer. In addition to design methods and theories, the designer’s skills and experience in optical system design are particularly important, which may require years of practice to learn. The diversity and variety of results are limited because of the difficulty, time, and labor costs required. In this article, we propose an automatic design method for freeform optics that can achieve a diverse range of three-mirror designs. The optical specifications and the design constraints are the only inputs required, and a variety of results can be obtained automatically. The output results have various structures and various optical power distributions with high imaging qualities. By implementing the design method, designers can not only realize an overview of the solution space of the three-mirror freeform system, but can also focus on specific designs.

## Introduction

An optical system is an assembly of optical components that operate together to produce a desired function^1^. Optical design involves determining all the data required to describe an optical system, such as the shapes of the optical surfaces, the positions and sizes of the optical components, and the materials of the optical media^2^, so that massive light rays emerging from multiple objects reaches the as-designed targets perfectly. Early optical design studies focused on subjects such as ray-tracing methods and the theory of primary and higher-order aberrations, where strong skills and ingenuity were essential when performing the numerical calculations^3^. The design process begins by setting up a specific type of optical system and then finding a solution where some of the primary or higher-order aberrations are eliminated. The designer must then evaluate the system’s performance, make judicious changes, and then re-evaluate the design before making further changes; this process is repeated until a system that meets the design requirements is obtained^2^. Considerable effort was required from the lens designers to accomplish designs such as telescopic, microscopic, and photographic under the conditions available at that time.

In the 1950s, the arrival of electronic computers opened a new era in optical design. Initially, computers were used to perform ray tracing rapidly, calculate the aberrations and evaluate the designed system’s performance; later, nonlinear equations from aberration theories were solved using computers to eliminate some of the aberrations^4,5^. Nowadays, computer-aided design has become more powerful than ever and has greatly improved the efficiency and quality of optical design. After the merit functions and boundary conditions (constraints) are set, the coefficients for each component in the system are then obtained by minimizing the merit functions using optimization algorithms; this process is simply called optimization^2,6,7^. Optical design through optimization is both an art and a science, and successful designs are believed to have to be accomplished under the guidance of a designer^7,8^. Optimization requires one (or a series of) initial solution(s) that are provided by the designer, and the variety of the optimization results obtained is limited by the initial solution(s)^2,7^. Optimization is an iterative process that combines evaluation and modification and the speed and effectiveness of the process are strongly dependent on the strategies adopted. To reduce the blindness involved in the optimization process, designers attempt to judge the system’s potential based on having achieved the expected requirements during the design process, but this judgment may be inaccurate and good results can be lost, potentially causing much better solutions to be ignored.

Since the introduction of computer-aided optical design, and despite the increasing degree of automation, design without human interaction is generally considered to be impossible. An initial solution must be provided and the optimization must then be performed by designers^2,6–10^. However, we may imagine that optical design in the future will have the following three characteristics: (1) Human operators will not participate in the design process and will not need to make decisions during the design. Only primary knowledge about optical design is required and the designer will only need to provide the system specifications and constraints. (2) All output results will satisfy the specifications and constraints given, and the imaging quality merits meet the given requirements. A variety of optical systems of different types will be obtained to provide an overview of the solution space of the optical system under the given specifications and constraints. (3) The main job of the designer will be to browse the output results, consider factors such as manufacturing and system structure comprehensively and select the preferred design.

An optical freeform surface is an optical surface that lacks rotational symmetry^11^. Freeform surfaces not only improve the overall performance of optical systems^12–14^ but also bring novel functions to these systems^15,16^. With advancements in optical processing and testing^17–19^, freeform surfaces are feasible in practical application. Freeform surfaces bring more degrees of freedom to optical systems and increase the dimensions of the aberration equations. There are three main ways to find an initial solution for a freeform system: an existing design, aberration theories^20,21^, and direct design methods^22–26^. In recent years, some procedures in the freeform system design process have been automated. Examples include improving the system’s imaging quality to achieve diffraction-limited performance^27^, varying the system structure^28^, determining initial solutions for four-mirror systems^29^, and exploring the solution space for freeform system design^30^.

In this work, towards realizing automatic optical design, we propose a result-diversified automatic design method for freeform optics. It is used to explore the solution space of three-mirror freeform systems and design imaging systems working in the visible (VIS) band and the long-wavelength infrared (LWIR) band. The designer must only provide the optical system specifications, such as the focal length, entrance pupil diameter, and full field-of-view angle, and input them to the computer. Through an automatic computer-based calculation process without human interaction, a variety of results that meet the design requirements are obtained that have various optical power (OP) distributions and various structures.

The proposed design method is composed of five phases, which is summarized as follows: (1) Construct a series of coaxial spherical systems with various OP distributions {***P***}: ***P***_**1**_, ***P***_**2**_, …, ***P***_***m***_, …, ***P***_***M***_. (2) For every coaxial spherical system in {***P***}, e.g., ***P***_***m***_, find out a series of unobscured systems that meet the given constraints $\left\{ {{\tilde{\boldsymbol C}}} \right\}_m:{\tilde{\boldsymbol C}}_{{\boldsymbol{m}},{\mathbf{1}}},{\tilde{\boldsymbol C}}_{{\boldsymbol{m}},{\mathbf{2}}}, \ldots ,{\tilde{\boldsymbol C}}_{{\boldsymbol{m}},t}, \ldots ,{\tilde{\boldsymbol C}}_{{\boldsymbol{m}},{\boldsymbol{T}}_{\boldsymbol{m}}}$. (3) Based on the unobscured off-axis systems contained in $\left\{ {{\tilde{\boldsymbol C}}} \right\}_m$, construct freeform systems and correct the OP of the entire system. Obtain freeform systems $\left\{ {{\tilde{\boldsymbol F}}} \right\}_m:{\tilde{\boldsymbol F}}_{{\boldsymbol{m,}}{\mathbf{1}}}{\mathrm{,}}{\tilde{\boldsymbol F}}_{{\boldsymbol{m,}}{\mathbf{2}}}{\mathrm{, \ldots ,}}{\tilde{\boldsymbol F}}_{{\boldsymbol{m,t}}}{\mathrm{, \ldots ,}}{\tilde{\boldsymbol F}}_{{\boldsymbol{m,T}}_{\boldsymbol{m}}}.$ (4) For every freeform system in the set $\left\{ {{\tilde{\boldsymbol F}}} \right\}_m$, improve the system imaging quality to its highest value by calculating the shape of each optical surface and finding the optimal tilt angle for the image plane. (5) Calculate the system imaging quality metric (or other evaluation metrics) and output the systems that meet the given requirements.

Following the procedures above, a program was developed using MATLAB and was finally deployed on a cluster system, the High-Performance Computation (HPC) platform of Tsinghua University. We had access to 50 computing nodes in the cluster system, each of which was a symmetrical multi-processing server composed of two six-core processors (2.93 GHz). In this work, only the metrics required to evaluate the system imaging quality were calculated using commercial software; the rest of the work, including ray tracing and calculation of the freeform surface shape was completed independently using the method proposed in this work.

## Results

### Example 1

The first example is a three-mirror freeform imaging system working in the LWIR band (8–14 μm). The full field-of-view angle is 8° × 6°. The focal length is 50 mm. The entrance pupil diameter is 27.78 mm. The F-number is 1.8. The primary system wavelength is 10 μm. After the computation is complete, 11 × 11 field points are sampled over the 8° × 6° field and the average of the root-mean-square values of the wavefront error (AVG WFE RMS) are calculated, which are used as the metric to evaluate the system performance. Systems with the AVG WFE RMS no greater than 0.075*λ*, where *λ* is the primary wavelength, are considered good results that meet the imaging quality requirements. These systems could be considered diffraction-limited or near-diffraction-limited. The computing task is completed in approximately 41.8 h without human interaction. A total of 127 freeform systems that satisfy the design requirements are obtained and the average time to obtain one system is 19.7 min.

**Fig. 1:**
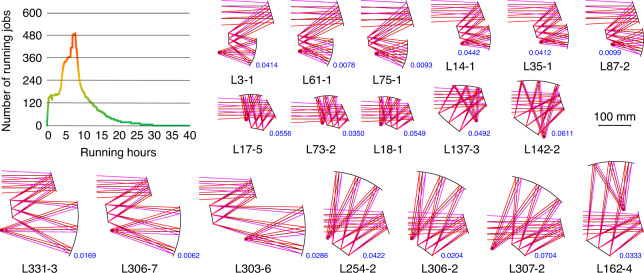
Curve of the number of running jobs versus the running time and part of the output results of design example 1. The number marked alongside the system layout is the AVG WFE RMS of the system with the unit of *λ* = 10 μm

In this work, the computing task for the design of a three-mirror freeform system consists of multiple independent computing jobs, each of which corresponds to the calculation of a freeform system ${\tilde{\boldsymbol F}}_{{\boldsymbol{m,t}}}$ that has the structure of ${\tilde{\boldsymbol C}}_{{\boldsymbol{m,t}}}$ with the OP distribution of ***P***_***m***_. The curve of the number of running jobs over the running time for design example 1 is shown in Fig. 1. The horizontal axis represents the running time (units: h), and the vertical axis represents the number of running jobs. By integrating the number of running jobs with respect to the running time and then distributing the integration result over 600 cores, a time of 6.33 h can be obtained, which can be used as a metric to evaluate the computing time. This shows that the actual computing time could be greatly reduced if an appropriate computing job management strategy is used or sufficient cores are occupied.

Figure 1 shows some of the design results on the same scale. Systems L3-1, L61-1, and L75-1 have similar structures and volumes but different OP distributions, as do systems L14-1, L35-1, and L87-2, which have more compact structures and smaller volumes. Systems L17-5, L73-2, and L18-1 have structures that are not commonly seen and are the most compact among the output results. Systems L137-3 and L142-2 have spherically-shaped envelopes. The primary mirror and the secondary mirror are convex in the systems in the final row (L331-3, L306-7, and L303-6; L254-2, L306-2, and L307-2), which leads to large system volumes. System L162-4 has a structure that has rarely been seen before. All the output results for design example 1 are presented in Fig. S1 and the imaging quality metrics are listed in Table S1.

**Fig. 2:**
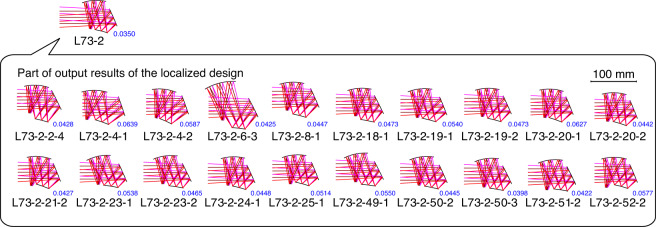
Part of the output results from the additional design in the localized solution space based on system L73-2 from design example 1. The number marked alongside the system layout is the AVG WFE RMS of the system with the unit of *λ* = 10 μm

The computation described above can be considered to be a coarse search of the solution space for the optical system. Next, an additional localized design can be carried out to obtain more design results, which is a fine search of the localized area of the solution space. The center of the fine search can either be systems that are diffraction-limited or have relatively high imaging qualities. For example, using system L73-2 in example 1 as the center, a smaller OP distribution range is determined, while the type of the structure is fixed and other computing specifications are maintained. The computing task is deployed on a computing workstation that has two 18-core processors (2.30 GHz). In a computation time of 39.13 h, a total of 59 freeform systems are obtained and the average time to obtain one system is 39.8 min, the structures of which are various. A portion of the output results is shown in Fig. 2, and all the output results are presented in Fig. S2 and the detailed imaging quality metrics are listed in Table S2.

### Example 2

**Fig. 3:**
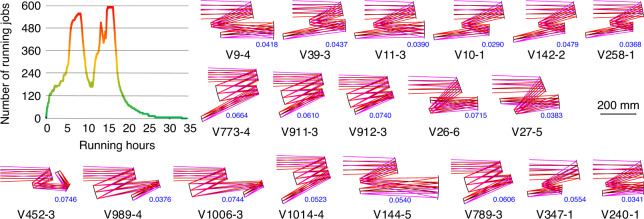
Curve of the number of running jobs versus the running time and part of the output results for design example 2. The number marked alongside the system layout is the AVG WFE RMS of the system with the unit of *λ* = 587.6 nm

The second example is a freeform imaging system working in the VIS band (420–680 nm). The object distance is infinite and the full field-of-view angle is 4° × 4°. The focal length is 450 mm. The entrance pupil diameter is 50 mm. The F-number is 9. The primary system wavelength is 587.6 nm. After 35.3 h of computation without human interaction, a total of 59 freeform systems are obtained and the average time to obtain one system is 35.9 min. The curve of the number of running jobs versus the running time is shown in Fig. 3. The theoretical computing time metric is 11.7 h for design example 2.

Figure 3 shows some of the output results on the same scale. Systems V9-4, V39-3, and V11-3 have the three typical structures contained among the output results. Systems V10-1, V142-2, and V258-1 all have the same structure but have different volumes. Systems V773-4, V911-3, and V912-3 have similar structures but have different image plane positions. Systems V26-6, V27-5, and V452-3 have structures that are rarely seen and the volume of system V452-3 is relatively small. Systems V989-4 and V1006-3 both contain small tertiary mirrors with short back working lengths. In system V1014-4, the primary mirror and the tertiary mirror are located close together and can be fabricated on the same substrate. In system V144-5, the structure could be folded by placing planar mirrors in the middle of the light path to allow the structure to be more compact. Systems V789-3, V347-1, and V240-1 have smaller volumes and compact structures. V240-1 is possibly the best design among all the output results. All the output results are presented in Fig. S3 and the imaging quality metrics are listed in Table S3.

At present, the majority of the computing time is spent in Phase 4 for all the design examples. When the program is deployed on the HPC cluster system, the time typically spent by each phase is as follows. Phase 1 and Phase 3 generally spend seconds of calculation, because Phase 1 calculates the surface curvature radii and surface distances targeting at the focal length and Phase 3 only involves the calculation of freeform shape of three mirrors. Phase 2 takes hours of calculation to perform an exhaustive search of possible unobscured systems, depending on the number of computing jobs and searching density. In Phase 5, it takes minutes of calculation by CODE V to evaluate the imaging qualities of the systems and output the good design results. Therefore, the bottleneck of the time consumption is in Phase 4.

## Discussion

The proposed design method has the following new features. It only needs system specifications as the only input, and does not need any initial design as the starting point. It can automatically distribute the OP among the mirrors in the system and automatically search for various structure forms of three-mirror system. It can provide a variety of high-quality system designs simultaneously by a coarse search on the solution space and can also focus on specific designs by a fine search on the localized solution space. Lastly, the grid search by this method is scalable and suitable for parallel computing acceleration. Optimization will only improve the system that is given to it and this system largely influences the optimization result. Local or global optimization algorithms starting with systems, for example, where only the first-order properties are fulfilled, will easily end up with unexpected results if no human interaction is involved. The automated design framework^27^ must require a planar system as the starting point, but can directly obtain a diffraction-limited system automatically. By contrast, the proposed method only requires the system specifications, and a variety of systems with different OP distributions and structures can be obtained by the coarse and fine search of the solution space.

The proposed design method provides new procedures of designing three-mirror freeform imaging system. Rather than spend time finding initial solutions and performing optimizations, the designer only needs to determine the optical specifications and constraints, and then input them into the computer and wait for the results to be outputted. The majority of the designer’s work will involve browsing through and analysing the multiple optical systems that are obtained and selecting the appropriate system as the final design. By using the automatic design method, we are more confident when selecting the system that meets our requirements most closely. For example, most of the systems have structures similar to L3-1 in Fig. 1 or V9-4 in Fig. 3, indicating that this type of structure is beneficial for achieving high imaging qualities under the given specifications. In design example 1, more than half of the systems have a convex primary mirror, which also leads to a large system volume. It is easy to find out which systems have smaller volumes than other designs, e.g., system L18-1 in design example 1 and system V240-1 in design example 2. Since various designs can be achieved and we can compare them with each other, the results of the proposed design method deliver information that other design methods cannot provide.

**Fig. 4:**
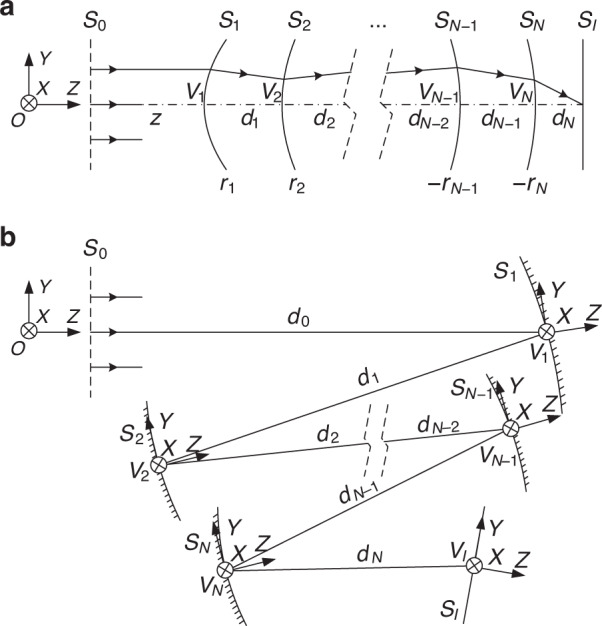
Coordinates and sign conventions in (**a**) coaxial and (**b**) off-axis optical systems

In an optical system, there are multiple coefficients that can be used as optimization variables, but these coefficients and the optical properties of the optical systems are not connected directly. Rather than use a grid search on these coefficients, the design method searches the structure of the system and the optical properties of individual components. The solution space of the three-mirror freeform system is discretized, and an optical system with a specific OP distribution and a specific structure is regarded as a grid point within the solution space. Each grid point represents an initial design for next phases and corresponds to a final freeform system which meets or does not meet the given quality requirement. Due to the high degree-of-freedom of freeform surfaces, the effective calculation of surface shapes, and the high-precision fitting method that considers the imaging requirements, systems with specific OP distributions and structure forms can achieve high imaging quality. As a result, a variety of design results with various OP distributions and various structures can be obtained, which represents a map of the solution space. Based on this solution map, the designers gain an overview of the solutions of three-mirror freeform system, which will be beneficial for selection of satisfactory results.

The amount of computation required is determined by (1) the dimensions of the variables of the solution space and (2) the number of OP distributions and structures. The first factor is determined by the number of components in the system, which is fixed in this work, and the second factor is determined by the range and density of the curvature radii of the surfaces and the surface distances. The amount of calculation increases exponentially as the number of components, the range, or the density of the coefficients increases. Thereby, the number of output results will be increased and the diversity of the results will be improved. However, because computing jobs are independent of each other, the running time could be reduced using parallel computation. Thus, the method is scalable, which means that it is applicable to large scale problems that contain vast numbers of computing jobs. As long as sufficient computing resources are provided, the proposed method remains feasible for design systems with larger numbers of optical surfaces and varied aperture stop positions, and the diversity and number of the output results could be increased. The current total computing time spent by the proposed method is long, but various design results are obtained and each design takes much less time on average. In addition, the whole design process by the proposed method does not need human involvement and thus human efforts would be saved by high-performance computer. For conventional design methods such as optimization, it will take days or months to complete hundreds of high-imaging optical systems with various structure forms and OP distributions.

When a greater variety of good results is required, one simple but effective option is to increase the density of the grid points. For example, in this work, Phase 2 performs an exhaustive search of the possible structure forms of the three-mirror system, but some possible structures are not shown up in the output result of the design example. By increasing the density and number of OP distributions and structures, more possible structures will emerge if they exist. However, there are numerous optical system solutions that meet the given design specifications and requirements and it is impossible to find out every solution. Furthermore, high density of searching grid points will result in a huge amount of computation, which is unnecessary because some of the good results are similar to each other in terms of OP distribution and structure and only appropriate grid point density is required for the different areas in the solution space. In order to balance the diversity of the output results with the amount of computation required in the design method, we can initially implement the design method using low-density grid points to obtain a series of results that are distributed widely over the solution space, which represents a coarse search of the solution space. Then, one can select several satisfactory designs from the output result of the coarse search and implement the design method using high-density grid points in a localized solution space to find more designs near the satisfactory designs above, which represents a fine search of the solution space.

The design results obtained by the coarse search can sometimes directly meet the design requirements, sometimes not, but in either case, a fine search can always be performed to find satisfactory solutions. For example, by using the proposed method, we design a system that has a field-of-view of 4° × 4°, a focal length of 600 mm and an entrance pupil of 200 mm, reference to the design reported in ref. ^21^. In the coarse search, systems with various OP distributions and structures are obtained, but no system has the AVG WFE RMS smaller than 0.075*λ*. However, a variety of designs that have relatively high imaging quality are obtained (see Fig. S4 and Table S4), with the AVG WFE RMS reaching down to 0.11*λ*. Taking one design as the center, the fine search is conducted and systems that meet the imaging quality standard are eventually obtained (see Fig. S5 and Table S5). The specific parameters used for the designs above are shown in Table S6.

In future development, several promising works related to the result-diversified automatic design method could be performed. Considering the manufacturability of freeform systems, the freeform departure and tolerances of every mirror and sensitivity of alignment in the system could be evaluated at different phases in the design process, and systems benefiting to fabrication and assembly can be obtained and selected. In addition to three-mirror freeform systems, the proposed method is feasible for other types of systems, because the calculating of surface shape is based on Fermat’s principle and the law of refraction and reflection, according to the object-image relationship of the system. After modifying the methods used in each phase, the method is feasible to design systems that have more or less than three surfaces, different aperture stop locations, or having lenses. The methods used in Phases 1 and 2 can be replaced with other advanced methods that are more effective in solving for first-order solutions and various unobscured structures. In Phases 3 and 4, methods other than the point-by-point method can be implemented to construct the freeform systems and improve their imaging qualities, as long as good imaging quality can be achieved automatically. To guarantee higher rates for derivation of good results in shorter computation times, it is worthwhile to study how to use aberration theory to direct determination of the OP distribution, the structure, and the aperture stop position. By deploying the result-diversified method on a sufficiently powerful computing system and modifying the program, massive design results with high imaging quality can be obtained in a few hours, which will be meaningful to both the engineering and research fields.

## Materials and methods

The coordinates and sign conventions are defined first. We initially define the global coordinates *O*-*XYZ*, as shown in Fig. 4a for a coaxial system and Fig. 4b for an off-axis system. The number of optical surfaces in the system is *N*. These optical surfaces are denoted by *S*_1_, *S*_2_, …, *S*_*i*_, …, *S*_*N*_. The system contains another two special surfaces, *S*_0_ and *S*_*I*_, where *S*_0_ is a virtual surface located in the front of the optical system and *S*_*I*_ is the image plane surface. Let the light rays in the central field (0°) travel along the direction of the unit axial vector ***OZ***. These light rays emerge from *S*_0_ and reach *S*_*I*_. As in the coaxial system shown in Fig. 4a, the system is rotationally symmetrical about the optical axis OZ. The center of the sphere with surface *S*_*i*_ is located on the optical axis and denoted by *O*_*i*_ (not marked in the figure)*. S*_*i*_ intersects the optical axis at the vertex point *V*_*i*_, as indicated in the figure. The radius of curvature of *S*_*i*_ is denoted by *r*_*i*_. When the vector ***V***_***i***_***O***_***i***_ and the unit axial vector ***OZ*** are oriented in the same direction, the sign of *r*_*i*_ is positive; otherwise, its sign is negative. The distance between surfaces *S*_*i*_ and *S*_*i*+1_ is denoted by *d*_*i*_, which is equal to the vector length |***V***_***i***_***V***_***i+*1**_|. When the vector and the unit axial vector ***OZ*** are oriented in the same direction, the sign of *d*_*i*_ is positive; otherwise, its sign is negative. In the off-axis system shown in Fig. 4b, in which there is no axis of rotational symmetry for the system, local coordinates must be set up at every optical surface and the surface shapes are described using these local coordinates. The chief ray (denoted by CR) of the central field is set as the reference in the off-axis systems. The point of incidence of the CR of the central field on surface *S*_*i*_ is denoted by *V*_*i*_. Local coordinates *V*_*i*_*-XYZ* are defined for surface *S*_*i*_, with the origin point being *V*_*i*_. The direction of the unit axial vector ***V***_***i***_***Z*** lies parallel to the direction of the normal vector at point *V*_*i*_ on surface *S*_*i*_. The unit axial vector ***V***_***i***_***Y*** lies parallel to the surface *O-YZ* and lies perpendicular to the vector ***V***_***i***_***Z***. The unit axial vector ***V***_***i***_***X*** is oriented in the same direction as the unit axial vector ***OX***. Specifically, the CR of the central field intersects surface *S*_0_ at point *V*_0_ and intersects surface *S*_*i*_ at *V*_*I*_. Unless otherwise stated, in the coordinates *V*_*I*_*-XYZ*, the unit axial vector ***V***_***I***_***Z*** lies perpendicular to the image plane; the unit axial vector ***V***_***I***_***Y*** is oriented parallel to the surface *O-YZ* and perpendicular to ***V***_***I***_***Z***; and the unit axial vector ***V***_***i***_***X*** is oriented in the same direction as the unit axial vector ***OX***.

**Fig. 5:**
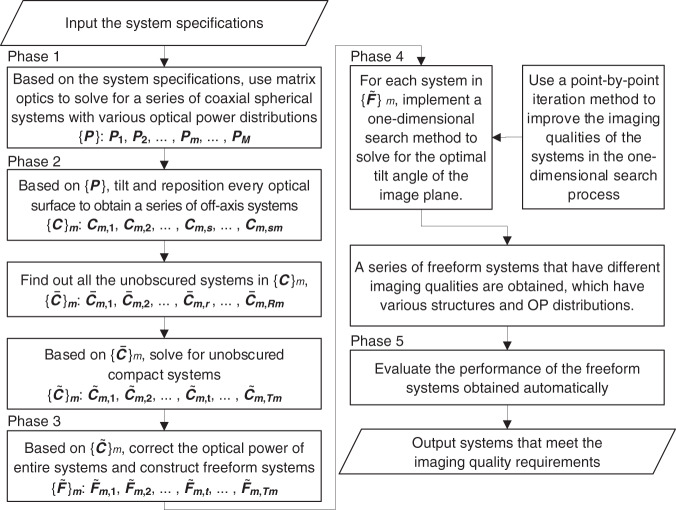
Flow chart of the key procedures of the proposed automatic design method

The flow chart of the key procedures of the proposed design method is shown in Fig. 5 and the detailed phases of this method are described as follows.

### Phase 1

Solve for a series of coaxial spherical systems with various OP distributions. Using the matrix approach for first-order optics, the reflection matrix *R*_*i*_ for reflection of the light ray at surface *S*_*i*_ is:

1 $$R_i = \left[ {\begin{array}{*{20}{c}} 1 & {\left( {n_i - n_{i - 1}} \right)/r_i} \\ 0 & 1 \end{array}} \right]$$

where *n*_*i*_ represents the refractive index of the medium between *S*_*i*_ and *S*_*i*+1_. The transfer matrix *D*_*i*_ of a light ray propagating from *S*_*i*_ to *S*_*i*+1_ is:

2 $$D_i = \left[ {\begin{array}{*{20}{c}} 1 & 0 \\ { - d_i/n_i} & 1 \end{array}} \right]$$

The system matrix for the complete system *T* is:

3 $$T = R_ND_{N - 1}R_{N - 1} \ldots R_2D_1R_1 = \left[ {\begin{array}{*{20}{c}} B & A \\ D & C \end{array}} \right]$$

where *A*, *B*, *C*, and *D* are functions of *r*_*i*_, *d*_*i*_, and *n*_*i*_. Therefore, the image focal length of the optical system can be obtained using *A*(*r*_*i*_, *d*_i_, *n*_*i*_) and is

4 $$f^{\prime} = \frac{{n_N}}{{A\left( {r_i,d_i,n_i} \right)}}$$

Because *f*′ is given, Eq. (4) represents the equation that the spherical curvature radii *r*_*i*_ (*i* = 1, 2, …, *N*) and surface distances *d*_*i*_ (*i* = 1, 2, …, *N*−1) must satisfy. In a reflection system, the condition *n*_*i*_ = −*n*_*i*−1_ applies; therefore, all the refractive indexes are canceled by each other. Eq. (4) has an infinite number of solutions and thus it is impossible to discuss all solutions to Eq. (4). Given that there are manufacturability limits in practice, some solutions to Eq. (4) should be disregarded and constraints should be set to narrow the solution space; this will be discussed in the following part.

In Eq. (4), there is a total of 2*N*−1 parameters for the radii of curvature and mirror distances, which are *r*_1_, *r*_2_, …, *r*_*N*_, and *d*_1_, *d*_2_, …, *d*_*N*−1_. As long as 2*N*−2 parameters out of the 2*N*−1 parameters are given, it is possible to solve for the last remaining parameter. After the 2*N*−1 parameters are obtained, an additional parameter *d*_*N*_ can be determined using first order optics (not shown in Eq. (4)), where *d*_*N*_ represents the distance between the last optical surface and the image plane. Therefore, there are a total of 2*N* parameters that describe the coaxial system. The 2*N* parameters are placed together in a row vector ***P*** = [*r*_1_, *r*_2_, …, *r*_*N*−1_, *r*_*N*_, *d*_1_, *d*_2_, …, *d*_*N*−1_, *d*_*N*_], which is used to represent a coaxial spherical system with a specific OP distribution. In this work, we assume that the given 2*N*−2 parameters are *r*_1_, *r*_2_, …, *r*_*N*−1_, *r*_*N*_, *d*_1_, *d*_2_, …, *d*_*N*−2_. Sequences of the radii of curvature *r*_*i*_ (*i* = 1, 2, …, *N*) are given as *r*_min_, *r*_min_+Δ*r*, *r*_min_+2Δ*r*, …, *r*_max_, with the range [*r*_min_, *r*_max_] and with interval Δ*r*. Sequences of mirror distances *d*_*i*_ (*i* = 1, 2, …, *N*−2) are given as *d*_min_, *d*_min_+Δ*d*, *d*_min_+2Δ*d*, …, *d*_max_, with the range [*d*_min_, *d*_max_] and the interval Δ*d*. For every combination of *r*_*i*_ (*i* = 1, 2, …, *N*) and *d*_*i*_ (*i* = 1, 2, …, *N*−2), the corresponding *d*_*N*−1_ can be solved using Eq. (4) and *d*_*N*_ can then be obtained using first-order optics. Following the procedure described above, with a series of 2*N* parameters obtained, a series of coaxial spherical systems with focal length *f*′ and various OP distributions are obtained and denoted by ***P***_**1**_, ***P***_**2**_, …, ***P***_***m***_, …, ***P***_***M***_; the set of these distributions is denoted by the symbol {***P***}.

As stated above, constraints are required to limit the range of values of some coefficients in the vector ***P***_***m***_. By changing the range of the radius of curvature *r*_*i*_ (*i* = 1, 2, …, *N*), the values of the radii of curvature and the positive/negative state of the OP of that optical surface can be controlled. In this work, the range for *r*_*i*_ is [−1000, 1000] (the units are millimeters hereinafter, unless otherwise stated). For the range of *d*_*i*_, three aspects must be considered. First, the values should not be too large to avoid large system volumes. Second, the values should not be too small or it may be impossible to ensure that the system is unobscured in the subsequent phases. Third, the differences between two arbitrary mirror distances should not be too large to guarantee system compactness. Because the overall optical system size is usually comparable to the entrance pupil size, we use the entrance pupil diameter (EPD) as the unit length to describe the range of mirror distances, e.g., EPD≤|*d*_1_|≤4 × EPD. When determining the interval values Δ*r* and Δ*d*, we must consider the balance between the computation time and the number of output results. If the values of Δ*r* and Δ*d* are too high, there will be fewer output results; however, values that are too small will increase the number of output results but will also consume more computation time.

### Phase 2

**Fig. 6:**
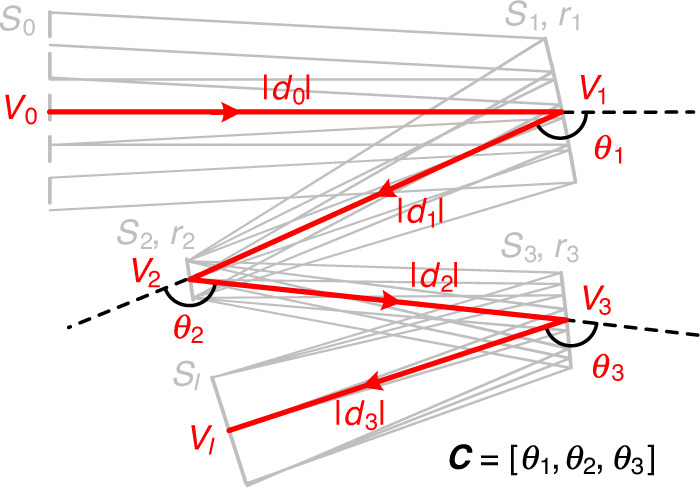
Notation to describe the structure of a three-mirror system

For each coaxial spherical system ***P***_***m***_ obtained in the previous step, tilt and reposition every surface in the system to obtain a series of noncoaxial systems while maintaining the direction of incidence of the CR of the central field in the object space. The systems obtained have various structures and can be considered to be field-biased, or off-axis, or a combination of the two. As shown in Fig. 6, where the three-mirror system is used as an example to explain the principle and the notation when solving for noncoaxial systems, the system corresponds to a coaxial system with a specific OP distribution ***P*** = [*r*_1_, *r*_2_, *r*_3_, *d*_1_, *d*_2_, *d*_3_]. Every mirror is tilted and repositioned by following the principle described below: the distances between the original points of the local coordinates are equal to the surface distances, which means that |***V***_**1**_***V***_**2**_| = *d*_1_, |***V***_**2**_***V***_**3**_| = *d*_2_, and |***V***_**3**_***V***_***I***_| = *d*_3_; at the same time, every mirror is tilted by a specific angle around the unit axial vector ***V***_***i***_***X*** in each local coordinate system.

For convenience of description, we use a vector ***C*** to represent the system structure. Noting that the path of the CR of the central field is a fold line that can be used to describe the system structure, we define ***C*** as a vector that contains the following two types of information: (1) the lengths of each segment of the fold line, which represents the absolute value of the surface distances |*d*_*i*_|; and (2) the angles between every adjacent pair of segments of the fold line. As shown in Fig. 6, the vector ***V***_***i*−1**_***V***_***i***_ rotates by an angle *θ*_*i*_ (*i* = 1, 2, …, *N*) to coincide with the vector ***V***_***i***_***V***_***i*+1**_. Therefore, *θ*_*i*_ represents the deflection angle of the CR of the central field at each mirror, which should have the range −360° < *θ*_*i*_ < 0° or 0° < *θ*_*i*_ < 360°. For clarity of description, the sign convention for *θ*_*i*_ is given as follows: if the vector ***V***_***i*−1**_***V***_***i***_ rotates clockwise to coincide with the vector ***V***_***i***_***V***_***i*+1**_, *θ*_*i*_ is negative; otherwise, *θ*_*i*_ is positive. Thus, as shown in Fig. 6, *θ*_1_ < 0, *θ*_2_ > 0, and *θ*_3_ < 0. The vector ***C*** can now be written as ***C*** = [*θ*_1_, |*d*_1_|, *θ*_2_, |*d*_2_|, … *θ*_*i*_, |*d*_*i*_|, …, *θ*_*N*_, |*d*_*N*_|]. For systems with the same OP distribution, the mirror distances are the same for the different structures; therefore, only the angles in the vector ***C*** are maintained, which make it look like ***C*** = [*θ*_1_, *θ*_2_, … *θ*_*i*_, …, *θ*_*N*_]. Specifically, the structure of the coaxial system is denoted by a symbol with a subscript of 0, i.e., ***C***_**0**_ = [*θ*_10_, *θ*_20_, *θ*_30_] = [−180, 180, −180].

In vector ***C***, when the values of the angles (*θ*_1_, *θ*_2_, … *θ*_*i*_, …, *θ*_*N*_) change continuously, the corresponding system structure also varies and may be obscured or unobscured. However, it is only when the angles vary within a specific range that the system remains unobscured. Because the system may contain different unobscured structure types, there may be multiple ranges within which *θ*_*i*_ can change in vector ***C***. In other words, the range of *θ*_*i*_ in ***C*** is not continuous to guarantee that the system is unobscured. To find as many unobscured structures as possible, as many systems with different structures as possible can be listed, regardless of whether they are obscured or not, and the obscured systems can then be filtered out.

As stated above, there are angle ranges of −360° < *θ*_*i*_ < 0° or 0° < *θ*_*i*_ < 360°. If two structures ***C*** and ***C***′ satisfy ***C*** =− ***C***′, then the two structures are identical. Therefore, in the case of repeated consideration of the same structure, the range for *θ*_1_ should be within (−180°, 0°), while the ranges of *θ*_*i*_ (*i* = 2, 3, …, *N*) should be within (−360°, 0°). In this work, the range of *θ*_1_ is given by (−180°, −120°]; the range for *θ*_2_ is [120°, 240°]; and the range for *θ*_3_ is [−240°, − 120°]. For the angle interval Δ*θ*, the diversity of the structures of the output results must be considered and balanced with the computation time. By following the above steps, with respect to the system with the OP distribution ***P***_***m***_, a series of spherical systems ${\boldsymbol{C}}_{{\boldsymbol{m,}}{\mathbf{1}}}{\mathrm{,}}{\boldsymbol{C}}_{{\boldsymbol{m,}}{\mathbf{2}}}{\mathrm{, \ldots ,}}{\boldsymbol{C}}_{{\boldsymbol{m,s}}}{\mathrm{, \ldots ,}}{\boldsymbol{C}}_{{\boldsymbol{m,S}}_{\boldsymbol{m}}}$ are obtained_,_ where the set of these systems is denoted by {***C***}_*m*_ with a total number of elements *S*_*m*_. Next, all the unobscured systems in each set {***C***}_*m*_ are found and denoted by ${\bar{\boldsymbol C}}_{{\boldsymbol{m,}}{\mathrm{1}}}{\mathrm{,}}{\bar{\boldsymbol C}}_{{\boldsymbol{m,}}{\mathrm{2}}}{\mathrm{, \ldots ,}}{\bar{\boldsymbol C}}_{{\boldsymbol{m,r}}}{\mathrm{, \ldots ,}}{\bar{\boldsymbol C}}_{{\boldsymbol{m,R}}_{\boldsymbol{m}}}$; the set of these systems is denoted by $\left\{ {{\bar {\boldsymbol{C}}}} \right\}_m$ with a total number of elements *R*_*m*_. When a variety of unobscured systems with various OP distributions and various structures has been obtained, we can proceed directly to the next step and construct the freeform systems.

In this phase, filters could be implemented on the system structure or the volume limit because the system structure and the OP distribution will only change slightly in the coming phases. The systems in set $\left\{ {{\bar {\boldsymbol{C}}}} \right\}_m$ can be classified into several categories based on the geometry of the system structure; in each category, the value of *θ*_*i*_ in vector ***C*** is varied. By defining the new vector $\Delta {\bar{\boldsymbol C}}_{{\boldsymbol{m,r}}} = {\bar{\boldsymbol C}}_{{\boldsymbol{m,r}}} - {\boldsymbol{C}}_{\mathbf{0}} = \left[ {\Delta \theta _1,\Delta \theta _2, \ldots \Delta \theta _i, \ldots ,\Delta \theta _N} \right]$, where ***C***_**0**_ represents the coaxial system structure, the geometry of the system structure is classified using the positive/negative sign of Δ*θ*_*i*_ in the vector $\Delta {\bar{\boldsymbol C}}_{{\boldsymbol{m,r}}}$. For each category of the system structure geometry, the absolute value of Δ*θ*_*i*_ can be regarded as a metric to evaluate the system compactness. A smaller absolute value of Δ*θ*_*i*_ represents a system with high compactness. In this work, for each category of the system structure geometry in the set $\left\{ {{\bar{\boldsymbol C}}} \right\}_m$, we obtain systems with compact structures by minimizing the absolute value of Δ*θ*_*i*_ in the vector $\Delta {\bar{\boldsymbol C}}_{{\boldsymbol{m,r}}}$. The systems obtained are denoted by ${\tilde{\boldsymbol C}}_{{\boldsymbol{m,}}{\mathbf{1}}}{\mathrm{,}}{\tilde{\boldsymbol C}}_{{\boldsymbol{m,}}{\mathbf{2}}}{\mathrm{, \ldots ,}}{\tilde{\boldsymbol C}}_{{\boldsymbol{m,t}}}{\mathrm{, \ldots ,}}{\tilde{\boldsymbol C}}_{{\boldsymbol{m,T}}_{\boldsymbol{m}}}$ and the set of these systems is denoted by $\left\{ {{\tilde{\boldsymbol C}}} \right\}_m$ with a total number of elements *T*_*m*_. In this phase, the system volume can be evaluated by calculating the volume of the space occupied by the light bundle and systems with volumes that exceed the limit can be removed from the set $\left\{ {{\tilde{\boldsymbol C}}} \right\}_m$.

### Phase 3

Construct freeform systems based on the systems in the set $\left\{ {{\tilde{\boldsymbol C}}} \right\}_m$. After the process to eliminate the obscured structures in phase 2, the OP of the entire system has been changed. In this phase, the freeform shapes for every optical surface are calculated by following the object-image relationship of the system, so that the OP of the entire system is corrected while the OP of each mirror is changed only slightly. Correction of the OP of the entire system should follow these principles: first, it must be realized automatically; second, the system structure must remain unchanged after the correction; third, the change in the OP of each mirror is small. Any method that satisfies these three rules can be implemented in this phase.

In this work, we use the point-by-point construction method for freeform systems^26^ to correct the OP of the entire system. The point-by-point construction method calculates freeform surface shapes based on feature light rays and feature data points. Feature light rays are defined at every field position and are located at different positions over the entrance pupil. Feature data points are defined as the intersection points of the feature light rays with the optical surfaces and contain the information of the point coordinates and the normal direction on the optical surface. Because the field-of-view angle for each feature light ray is known, and based on the object-image relationship that the system provides perfect imaging, corresponding image point coordinates (target image point coordinates) can be obtained on the image plane. When calculating the shape of the surface *S*_*i*_, intersection point coordinates on surface *S*_*i*−1_ and the propagation direction towards *S*_*i*_ are obtained for all feature light rays by real ray tracing. Starting from a given initial feature data point on surface *S*_*i*_, the next feature light ray is then determined. Based on the coordinates and the normals of the feature data points that have already been calculated, the corresponding feature data point’s coordinates are obtained via the nearest-ray algorithm^26^. Next, when the ideal image point coordinates and the corresponding feature data point coordinates on *S*_*i*_ are known, the direction in which the feature light ray leaves *S*_*i*_ can be resolved using Fermat’s principle; then, by knowing both the direction of incidence and direction of departure of the feature light ray on *S*_*i*_, the normal directions of the corresponding feature data points can be obtained based on the law of reflection. The procedures above are repeated until the coordinates and normals of all feature data points are solved. Finally, the mathematical expressions for the freeform surface *S*_*i*_ are obtained via a fitting method that considers both the coordinates and the normals of all feature data points^31^. In this work, XY polynomials with up to sixth order terms are used to describe the shape of the freeform surfaces. The results in the design examples show that the precision of this fitting method is high enough to achieve high imaging quality of diffraction-limited or near-diffraction-limited. Following the procedures above, the shapes of the freeform surfaces in the system are all calculated in a given order (e.g., tertiary-secondary-primary mirrors) and the construction of the freeform system is completed.

By implementing the method described above, the following series of freeform systems is obtained: ${\tilde{\boldsymbol F}}_{{\boldsymbol{m,}}{\mathbf{1}}},{\tilde{\boldsymbol F}}_{{\boldsymbol{m,}}{\mathbf{2}}}, \ldots ,{\tilde{\boldsymbol F}}_{{\boldsymbol{m,t}}}, \ldots ,{\tilde{\boldsymbol F}}_{{\boldsymbol{m,T}}_{\boldsymbol{m}}}$, corresponding to the systems ${\tilde{\boldsymbol C}}_{{\boldsymbol{m,}}{\mathbf{1}}},{\tilde{\boldsymbol C}}_{{\boldsymbol{m,}}{\mathbf{2}}}, \ldots ,{\tilde{\boldsymbol C}}_{{\boldsymbol{m,t}}}, \ldots ,{\tilde{\boldsymbol C}}_{{\boldsymbol{m,T}}_{\boldsymbol{m}}}$. The set of freeform systems obtained is denoted by $\left\{ {{\tilde{\boldsymbol F}}} \right\}_{\it{m}}$.

### Phase 4

Improve the imaging quality of the freeform system. By following the phases above, a series of unobscured freeform systems $\left\{ {{\tilde{\boldsymbol F}}} \right\}_{\it{m}}$ with various OP distributions and structures has been obtained, but the system imaging qualities still require improvement. The method used in this phase must satisfy the three principles stated in Phase 3. In this work, we use a point-by-point iteration method to improve the imaging quality of the freeform systems^26^.

The point-by-point iteration method is the same as the construction method from the perspective that the shape of each optical surface is resolved by following the object-image relationship, which is also based on the feature light rays and feature data points. The difference is that, during the iteration process, the feature data point coordinates on *S*_*i*_ are obtained and retained by tracing the feature light rays incident on *S*_*i*_, while the surface normals are newly solved. When the traced data point coordinates and newly solved normal directions are known, a new freeform surface can be obtained by fitting.

In the point-by-point iteration method, an iteration round consists of calculation of the shapes of all optical surfaces in the system in a given order. Multiple iteration rounds can be performed until the imaging quality reaches the required value or it stops improving. In this work, the root-mean-square (RMS) values of the distances between the actual imaging points and the target imaging points are calculated at different field points and the average value of these distances (denoted by *σ*) is used as the metric to evaluate the imaging quality of the result of each round of iteration. As the iteration proceeds, the value of *σ* decreases and then gradually converges. When *σ* is smaller than a specified threshold *σ*_itr_, the iteration process is terminated. The rate at which this value decreases after each round of iteration, *τ*, is defined as *τ* = |*σ*′−*σ*|/*σ*, where *σ*′ and *σ* are used to evaluate the imaging qualities of the results of the previous and current rounds of iterations, respectively. When *τ* decreases below a specific threshold *τ*_itr_, the iteration process is terminated.

After an inspection of all available degrees-of-freedom for design of the freeform systems, we found that the tilt angle of the image plane has not been considered yet; therefore, the optimal tilt angle of the image plane must be determined to achieve the best possible imaging quality for the optical system. In this work, a one-dimensional search process is implemented. The image plane tilt angle is defined by the angle between the *Y* axis of the local coordinates and the *Y* axis of the global coordinates and denoted by *β*. In particular, when the CR of the central field is perpendicular to the image plane, the image plane tilt angle is denoted by *β*_0_. In a round of one-dimensional searching, a series of freeform systems with different image plane tilt angles is iterated to improve the system imaging quality until the iteration stops. In the first round of one-dimensional searching, the image plane tilt angles are given by a sequence varying within the range [*β*_0_*−β*_r_, *β*_0_+*β*_r_] with the interval Δ*β*. In the subsequent round of one-dimensional searching, the image plane tilt angles are given by a sequence varying within the range [*β*_opt_*−β*_r_, *β*_opt_+*β*_r_] with the interval Δ*β*, where *β*_opt_ is the image plane tilt angle of the system with the best imaging quality from the previous round of searching. As multiple rounds of one-dimensional searching are performed, the system’s imaging quality improves and the imaging quality metric *σ* converges. When *σ* is smaller than the specified threshold *σ*_srh_, the search process is terminated. When the improvement rate *τ* = (*σ*′*−σ*)/*σ* is lower than the specified threshold *τ*_srh_, the search process is terminated. *σ*′ and *σ* above are the imaging quality metrics for the results of the previous and current rounds of searching, respectively.

### Phase 5

By following the steps above, a series of freeform systems with various structures and various OP distributions is obtained. For each freeform system obtained, the imaging quality metrics are calculated, including the spot diameter of the imaging points, the modulation transfer function, and the RMS WFE over the field. In this work, systems where the AVG RMS WFE is lower than 0.075*λ* are eventually presented to the designers as the output results. The designers can then analyse the systems obtained and select their preferred designs. The specific parameters used for the design example 1 and 2 are shown in Table S7. The framework for the automatic design method is shown in Fig. S6.

## Supplementary information

Supplementary Information for Towards automatic freeform optics design: coarse and fine search of the three-mirror solution space
