# Supplementary material for: Towards automatic freeform optics design: coarse and fine search of the three-mirror solution space
Source: Light Sci Appl. 2021 Mar 29;10:65. doi: 10.1038/s41377-021-00510-z (PMC8005493; doi:10.1038/s41377-021-00510-z)

Supplementary Information for

Towards automatic freeform optics design: coarse and fine search of the three-mirror solution space

Benqi Zhang, Guofan Jin, and Jun Zhu*

State Key Laboratory of Precision Measurement Technology and Instruments, Department of Precision Instrument, Tsinghua University, Beijing 100084, China

*j_zhu@tsinghua.edu.cn

**Figure S1.** All output results of design example 1, which have the AVG WFE RMS smaller than 0.075 *λ*.








**Table S1.** Minimum, maximum, average, and standard deviation RMS values of the wavefront error across the field-of-view and the distortion percentage (maximum) of all output results of design example 1.

| System number: L#M-#N | | Minimum | Maximum | Average | Standard Deviation | Distortion Percentage |
| --- | --- | --- | --- | --- | --- | --- |
| M | N |  |  |  |  |  |
| 3 | 1 | 0.0166 | 0.0590 | 0.0414 | 0.0129 | 4.92 |
| 14 | 1 | 0.0135 | 0.0699 | 0.0442 | 0.0134 | 3.09 |
| 17 | 5 | 0.0200 | 0.0966 | 0.0556 | 0.0194 | 2.62 |
| 18 | 1 | 0.0243 | 0.0958 | 0.0549 | 0.0190 | 2.18 |
| 21 | 1 | 0.0149 | 0.1084 | 0.0569 | 0.0245 | 2.94 |
| 21 | 2 | 0.0164 | 0.0673 | 0.0444 | 0.0124 | 2.48 |
| 25 | 1 | 0.0090 | 0.0365 | 0.0252 | 0.0087 | 5.45 |
| 25 | 2 | 0.0113 | 0.0267 | 0.0198 | 0.0045 | 3.05 |
| 29 | 1 | 0.0188 | 0.0506 | 0.0381 | 0.0095 | 3.86 |
| 35 | 1 | 0.0140 | 0.0699 | 0.0412 | 0.0122 | 2.58 |
| 38 | 4 | 0.0156 | 0.0673 | 0.0429 | 0.0127 | 2.54 |
| 40 | 1 | 0.0341 | 0.1244 | 0.0706 | 0.0257 | 2.62 |
| 41 | 2 | 0.0169 | 0.1102 | 0.0585 | 0.0246 | 2.28 |
| 44 | 1 | 0.0050 | 0.0212 | 0.0143 | 0.0048 | 2.68 |
| 48 | 1 | 0.0075 | 0.0283 | 0.0166 | 0.0052 | 2.32 |
| 48 | 2 | 0.0112 | 0.0378 | 0.0275 | 0.0073 | 2.70 |
| 51 | 1 | 0.0145 | 0.0718 | 0.0473 | 0.0169 | 4.87 |
| 56 | 1 | 0.0098 | 0.0664 | 0.0416 | 0.0138 | 3.25 |
| 56 | 2 | 0.0096 | 0.0751 | 0.0413 | 0.0155 | 2.02 |
| 56 | 3 | 0.0058 | 0.0552 | 0.0334 | 0.0119 | 2.39 |
| 59 | 1 | 0.0139 | 0.1022 | 0.0541 | 0.0229 | 2.22 |
| 59 | 2 | 0.0180 | 0.1004 | 0.0538 | 0.0225 | 2.10 |
| 61 | 1 | 0.0025 | 0.0120 | 0.0078 | 0.0024 | 1.58 |
| 64 | 1 | 0.0067 | 0.0232 | 0.0161 | 0.0048 | 2.38 |
| 66 | 1 | 0.0082 | 0.0448 | 0.0292 | 0.0109 | 4.29 |
| 69 | 1 | 0.0171 | 0.0928 | 0.0600 | 0.0215 | 4.13 |
| 71 | 1 | 0.0395 | 0.0956 | 0.0648 | 0.0126 | 2.01 |
| 73 | 2 | 0.0063 | 0.0620 | 0.0350 | 0.0137 | 2.11 |
| 75 | 1 | 0.0038 | 0.0138 | 0.0093 | 0.0027 | 1.37 |
| 77 | 1 | 0.0048 | 0.0215 | 0.0131 | 0.0047 | 2.76 |
| 79 | 1 | 0.0032 | 0.0154 | 0.0092 | 0.0032 | 1.89 |
| 87 | 2 | 0.0040 | 0.0155 | 0.0099 | 0.0031 | 1.35 |
| 89 | 4 | 0.0045 | 0.0166 | 0.0103 | 0.0030 | 1.67 |
| 91 | 2 | 0.0066 | 0.0362 | 0.0239 | 0.0087 | 3.38 |
| 96 | 3 | 0.0037 | 0.0194 | 0.0127 | 0.0044 | 1.16 |
| 97 | 4 | 0.0112 | 0.0283 | 0.0181 | 0.0045 | 2.78 |
| 137 | 3 | 0.0262 | 0.0904 | 0.0492 | 0.0134 | 2.61 |
| 141 | 3 | 0.0358 | 0.0986 | 0.0550 | 0.0138 | 2.06 |
| 142 | 2 | 0.0356 | 0.1157 | 0.0611 | 0.0162 | 2.09 |
| 162 | 4 | 0.0152 | 0.0551 | 0.0333 | 0.0117 | 3.25 |
| 172 | 8 | 0.0308 | 0.0577 | 0.0443 | 0.0068 | 3.92 |
| 173 | 6 | 0.0147 | 0.0787 | 0.0559 | 0.0160 | 2.90 |
| 174 | 6 | 0.0245 | 0.0639 | 0.0438 | 0.0079 | 3.20 |
| 175 | 5 | 0.0429 | 0.1120 | 0.0692 | 0.0135 | 3.92 |
| 184 | 7 | 0.0139 | 0.0575 | 0.0329 | 0.0103 | 3.92 |
| 185 | 7 | 0.0181 | 0.0434 | 0.0291 | 0.0064 | 2.94 |
| 186 | 7 | 0.0168 | 0.0437 | 0.0301 | 0.0075 | 3.31 |
| 187 | 8 | 0.0125 | 0.0460 | 0.0312 | 0.0088 | 3.10 |
| 188 | 5 | 0.0341 | 0.0583 | 0.0429 | 0.0066 | 4.23 |
| 189 | 4 | 0.0092 | 0.0693 | 0.0451 | 0.0151 | 3.28 |
| 193 | 6 | 0.0107 | 0.0471 | 0.0309 | 0.0106 | 2.78 |
| 194 | 8 | 0.0126 | 0.0414 | 0.0235 | 0.0069 | 3.06 |
| 195 | 8 | 0.0134 | 0.0404 | 0.0239 | 0.0073 | 3.50 |
| 196 | 6 | 0.0092 | 0.0459 | 0.0208 | 0.0087 | 3.25 |
| 197 | 8 | 0.0196 | 0.0314 | 0.0239 | 0.0026 | 3.09 |
| 198 | 7 | 0.0072 | 0.0411 | 0.0272 | 0.0087 | 2.73 |
| 199 | 6 | 0.0106 | 0.0564 | 0.0349 | 0.0096 | 4.05 |
| 201 | 8 | 0.0101 | 0.0477 | 0.0298 | 0.0108 | 3.66 |
| 202 | 6 | 0.0082 | 0.0349 | 0.0223 | 0.0078 | 3.13 |
| 203 | 5 | 0.0144 | 0.0412 | 0.0263 | 0.0083 | 2.64 |
| 204 | 6 | 0.0196 | 0.0330 | 0.0248 | 0.0029 | 3.48 |
| 219 | 7 | 0.0089 | 0.0288 | 0.0205 | 0.0057 | 2.98 |
| 222 | 8 | 0.0128 | 0.0383 | 0.0251 | 0.0078 | 3.23 |
| 225 | 7 | 0.0142 | 0.0472 | 0.0320 | 0.0087 | 3.34 |
| 226 | 8 | 0.0167 | 0.0738 | 0.0469 | 0.0148 | 3.93 |
| 227 | 8 | 0.0105 | 0.0592 | 0.0370 | 0.0129 | 4.05 |
| 228 | 7 | 0.0110 | 0.0352 | 0.0222 | 0.0066 | 3.26 |
| 229 | 7 | 0.0082 | 0.0327 | 0.0225 | 0.0070 | 3.13 |
| 230 | 7 | 0.0074 | 0.0363 | 0.0226 | 0.0083 | 3.70 |
| 231 | 8 | 0.0088 | 0.0356 | 0.0229 | 0.0073 | 3.05 |
| 232 | 3 | 0.0161 | 0.0340 | 0.0247 | 0.0048 | 3.80 |
| 234 | 6 | 0.0045 | 0.0251 | 0.0151 | 0.0066 | 1.84 |
| 235 | 7 | 0.0055 | 0.0266 | 0.0145 | 0.0050 | 3.26 |
| 254 | 1 | 0.0222 | 0.0542 | 0.0403 | 0.0099 | 1.95 |
| 254 | 2 | 0.0208 | 0.0583 | 0.0422 | 0.0092 | 2.44 |
| 254 | 7 | 0.0040 | 0.0223 | 0.0125 | 0.0051 | 2.49 |
| 255 | 2 | 0.0114 | 0.0628 | 0.0439 | 0.0115 | 2.38 |
| 255 | 5 | 0.0062 | 0.0222 | 0.0157 | 0.0050 | 3.62 |
| 257 | 9 | 0.0082 | 0.0310 | 0.0205 | 0.0063 | 2.87 |
| 258 | 6 | 0.0087 | 0.0390 | 0.0253 | 0.0083 | 3.35 |
| 259 | 3 | 0.0324 | 0.0782 | 0.0560 | 0.0131 | 2.37 |
| 259 | 7 | 0.0150 | 0.0460 | 0.0305 | 0.0088 | 3.59 |
| 260 | 1 | 0.0204 | 0.0710 | 0.0537 | 0.0127 | 2.23 |
| 260 | 8 | 0.0047 | 0.0219 | 0.0151 | 0.0050 | 3.03 |
| 262 | 1 | 0.0180 | 0.0398 | 0.0298 | 0.0064 | 1.95 |
| 262 | 5 | 0.0037 | 0.0168 | 0.0111 | 0.0031 | 2.48 |
| 280 | 7 | 0.0091 | 0.0244 | 0.0161 | 0.0039 | 2.29 |
| 281 | 4 | 0.0078 | 0.0309 | 0.0179 | 0.0065 | 3.37 |
| 282 | 1 | 0.0052 | 0.0135 | 0.0078 | 0.0017 | 2.09 |
| 282 | 2 | 0.0058 | 0.0251 | 0.0126 | 0.0042 | 1.91 |
| 282 | 6 | 0.0064 | 0.0187 | 0.0122 | 0.0039 | 1.89 |
| 284 | 7 | 0.0058 | 0.0562 | 0.0224 | 0.0128 | 4.06 |
| 285 | 5 | 0.0078 | 0.0301 | 0.0165 | 0.0060 | 3.55 |
| 286 | 2 | 0.0261 | 0.0510 | 0.0408 | 0.0077 | 1.04 |
| 286 | 3 | 0.0063 | 0.0162 | 0.0109 | 0.0028 | 0.97 |
| 286 | 7 | 0.0045 | 0.0106 | 0.0075 | 0.0017 | 2.06 |
| 303 | 6 | 0.0101 | 0.0513 | 0.0286 | 0.0103 | 4.42 |
| 304 | 6 | 0.0119 | 0.0345 | 0.0210 | 0.0060 | 3.67 |
| 305 | 4 | 0.0067 | 0.0256 | 0.0151 | 0.0049 | 3.10 |
| 305 | 5 | 0.0333 | 0.0672 | 0.0513 | 0.0077 | 3.85 |
| 306 | 2 | 0.0143 | 0.0381 | 0.0204 | 0.0051 | 3.05 |
| 306 | 7 | 0.0023 | 0.0101 | 0.0062 | 0.0019 | 2.32 |
| 307 | 2 | 0.0309 | 0.1337 | 0.0704 | 0.0261 | 2.56 |
| 308 | 4 | 0.0050 | 0.0172 | 0.0106 | 0.0030 | 2.63 |
| 309 | 3 | 0.0221 | 0.1010 | 0.0615 | 0.0136 | 2.00 |
| 309 | 4 | 0.0105 | 0.0230 | 0.0135 | 0.0024 | 1.48 |
| 309 | 6 | 0.0030 | 0.0095 | 0.0063 | 0.0015 | 2.46 |
| 310 | 2 | 0.0152 | 0.0538 | 0.0341 | 0.0099 | 2.29 |
| 328 | 6 | 0.0109 | 0.0411 | 0.0268 | 0.0089 | 4.31 |
| 329 | 4 | 0.0068 | 0.0425 | 0.0233 | 0.0096 | 4.27 |
| 329 | 5 | 0.0403 | 0.0701 | 0.0495 | 0.0053 | 1.76 |
| 330 | 1 | 0.0066 | 0.0139 | 0.0099 | 0.0016 | 1.75 |
| 330 | 4 | 0.0301 | 0.0591 | 0.0470 | 0.0077 | 1.60 |
| 330 | 5 | 0.0179 | 0.0419 | 0.0294 | 0.0057 | 2.40 |
| 331 | 3 | 0.0072 | 0.0263 | 0.0169 | 0.0042 | 2.69 |
| 332 | 5 | 0.0081 | 0.0273 | 0.0158 | 0.0051 | 3.19 |
| 333 | 4 | 0.0055 | 0.0148 | 0.0099 | 0.0023 | 1.75 |
| 334 | 3 | 0.0137 | 0.0565 | 0.0348 | 0.0095 | 2.48 |
| 334 | 4 | 0.0077 | 0.0228 | 0.0139 | 0.0038 | 3.30 |
| 352 | 6 | 0.0130 | 0.0463 | 0.0310 | 0.0091 | 3.64 |
| 353 | 4 | 0.0077 | 0.0348 | 0.0223 | 0.0080 | 4.12 |
| 354 | 3 | 0.0061 | 0.0257 | 0.0144 | 0.0052 | 3.99 |
| 355 | 3 | 0.0043 | 0.0204 | 0.0107 | 0.0041 | 2.78 |
| 356 | 5 | 0.0036 | 0.0195 | 0.0117 | 0.0046 | 2.91 |
| 356 | 6 | 0.0278 | 0.0633 | 0.0448 | 0.0071 | 1.95 |
| 357 | 3 | 0.0205 | 0.0654 | 0.0438 | 0.0122 | 2.04 |
| 357 | 4 | 0.0028 | 0.0197 | 0.0112 | 0.0036 | 2.13 |

**Figure S2.** All output results of the additional design on the localized solution space of design example 1, which have the AVG WFE RMS smaller than 0.075 *λ*.


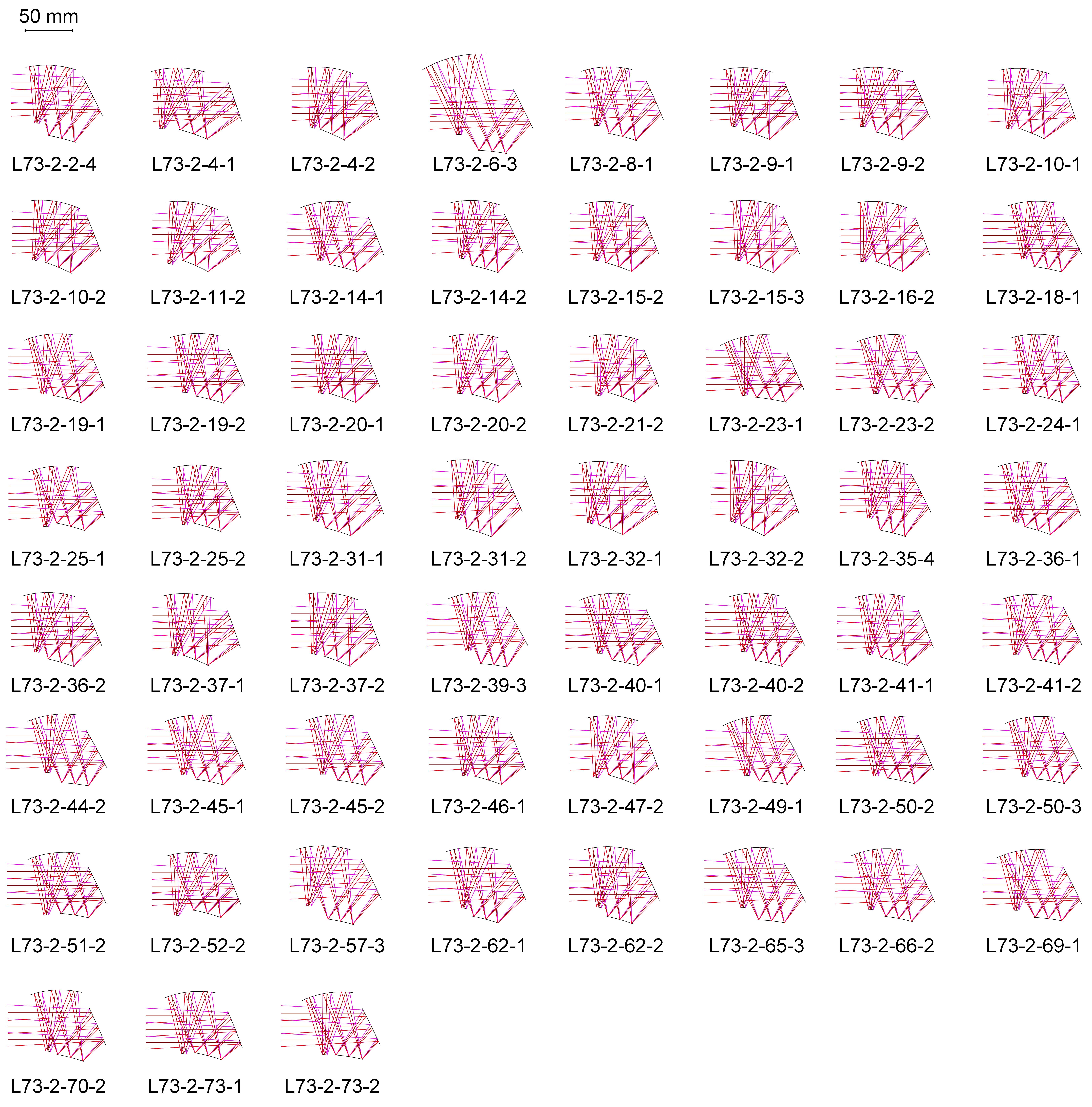


**Table S2.** Minimum, maximum, average, and standard deviation RMS values of the wavefront error across the field-of-view and the distortion percentage (maximum) of all output results of the additional design on the localized solution space of design example 1.

| System number: L73-2-#M-#N | | Minimum | Maximum | Average | Standard Deviation | Distortion Percentage |
| --- | --- | --- | --- | --- | --- | --- |
| M | N |  |  |  |  |  |
| 2 | 4 | 0.0124 | 0.0661 | 0.0428 | 0.0132 | 2.84 |
| 4 | 1 | 0.0192 | 0.1088 | 0.0639 | 0.0227 | 3.18 |
| 4 | 2 | 0.0343 | 0.1002 | 0.0587 | 0.0191 | 1.86 |
| 6 | 3 | 0.0237 | 0.0626 | 0.0425 | 0.0098 | 2.57 |
| 8 | 1 | 0.0125 | 0.0747 | 0.0447 | 0.0148 | 2.75 |
| 9 | 1 | 0.0223 | 0.0998 | 0.0548 | 0.0216 | 2.43 |
| 9 | 2 | 0.0123 | 0.0699 | 0.0447 | 0.0141 | 2.76 |
| 10 | 1 | 0.0246 | 0.1000 | 0.0554 | 0.0209 | 2.29 |
| 10 | 2 | 0.0093 | 0.0933 | 0.0492 | 0.0211 | 2.51 |
| 11 | 2 | 0.0239 | 0.0973 | 0.0531 | 0.0207 | 2.52 |
| 14 | 1 | 0.0355 | 0.1117 | 0.0659 | 0.0218 | 2.37 |
| 14 | 2 | 0.0156 | 0.0867 | 0.0479 | 0.0185 | 2.41 |
| 15 | 2 | 0.0178 | 0.0966 | 0.0511 | 0.0217 | 2.24 |
| 15 | 3 | 0.0171 | 0.0802 | 0.0461 | 0.0158 | 2.49 |
| 16 | 2 | 0.0122 | 0.0737 | 0.0428 | 0.0157 | 2.56 |
| 18 | 1 | 0.0125 | 0.0855 | 0.0473 | 0.0181 | 2.37 |
| 19 | 1 | 0.0120 | 0.0889 | 0.0540 | 0.0181 | 2.15 |
| 19 | 2 | 0.0097 | 0.0879 | 0.0473 | 0.0197 | 2.27 |
| 20 | 1 | 0.0258 | 0.1115 | 0.0627 | 0.0227 | 2.04 |
| 20 | 2 | 0.0075 | 0.0803 | 0.0442 | 0.0178 | 2.38 |
| 21 | 2 | 0.0090 | 0.0823 | 0.0427 | 0.0193 | 2.15 |
| 23 | 1 | 0.0104 | 0.0966 | 0.0538 | 0.0209 | 2.42 |
| 23 | 2 | 0.0157 | 0.0724 | 0.0465 | 0.0137 | 2.43 |
| 24 | 1 | 0.0112 | 0.0841 | 0.0448 | 0.0189 | 2.07 |
| 25 | 1 | 0.0166 | 0.1018 | 0.0514 | 0.0243 | 2.22 |
| 25 | 2 | 0.0177 | 0.1024 | 0.0505 | 0.0250 | 1.99 |
| 31 | 1 | 0.0072 | 0.0596 | 0.0364 | 0.0121 | 2.21 |
| 31 | 2 | 0.0052 | 0.0683 | 0.0397 | 0.0152 | 2.18 |
| 32 | 1 | 0.0237 | 0.0836 | 0.0478 | 0.0167 | 1.85 |
| 32 | 2 | 0.0366 | 0.0956 | 0.0609 | 0.0161 | 2.20 |
| 35 | 4 | 0.0338 | 0.1057 | 0.0666 | 0.0183 | 2.49 |
| 36 | 1 | 0.0085 | 0.0760 | 0.0431 | 0.0168 | 2.10 |
| 36 | 2 | 0.0105 | 0.0574 | 0.0359 | 0.0103 | 2.59 |
| 37 | 1 | 0.0265 | 0.0903 | 0.0522 | 0.0182 | 1.98 |
| 37 | 2 | 0.0102 | 0.0514 | 0.0338 | 0.0098 | 2.68 |
| 39 | 3 | 0.0107 | 0.0653 | 0.0408 | 0.0123 | 2.65 |
| 40 | 1 | 0.0081 | 0.0647 | 0.0393 | 0.0137 | 2.51 |
| 40 | 2 | 0.0096 | 0.0919 | 0.0507 | 0.0199 | 2.30 |
| 41 | 1 | 0.0194 | 0.0970 | 0.0523 | 0.0215 | 2.36 |
| 41 | 2 | 0.0151 | 0.0632 | 0.0434 | 0.0117 | 3.21 |
| 44 | 2 | 0.0131 | 0.0757 | 0.0431 | 0.0156 | 2.72 |
| 45 | 1 | 0.0083 | 0.0800 | 0.0451 | 0.0180 | 2.38 |
| 45 | 2 | 0.0120 | 0.0744 | 0.0455 | 0.0150 | 2.82 |
| 46 | 1 | 0.0241 | 0.0958 | 0.0519 | 0.0206 | 2.17 |
| 47 | 2 | 0.0064 | 0.0703 | 0.0385 | 0.0163 | 2.38 |
| 49 | 1 | 0.0237 | 0.1021 | 0.0550 | 0.0227 | 2.49 |
| 50 | 2 | 0.0081 | 0.0837 | 0.0445 | 0.0196 | 2.29 |
| 50 | 3 | 0.0118 | 0.0695 | 0.0398 | 0.0148 | 2.42 |
| 51 | 2 | 0.0080 | 0.0762 | 0.0422 | 0.0167 | 2.53 |
| 52 | 2 | 0.0243 | 0.1101 | 0.0577 | 0.0248 | 2.75 |
| 57 | 3 | 0.0089 | 0.0619 | 0.0345 | 0.0117 | 3.85 |
| 62 | 1 | 0.0222 | 0.0856 | 0.0527 | 0.0158 | 2.05 |
| 62 | 2 | 0.0313 | 0.0925 | 0.0583 | 0.0152 | 2.09 |
| 65 | 3 | 0.0084 | 0.0527 | 0.0348 | 0.0110 | 2.96 |
| 66 | 2 | 0.0107 | 0.0484 | 0.0309 | 0.0084 | 2.89 |
| 69 | 1 | 0.0090 | 0.0883 | 0.0480 | 0.0199 | 3.25 |
| 70 | 2 | 0.0188 | 0.0804 | 0.0440 | 0.0175 | 1.99 |
| 73 | 1 | 0.0171 | 0.0963 | 0.0519 | 0.0213 | 2.23 |
| 73 | 2 | 0.0072 | 0.0538 | 0.0314 | 0.0118 | 2.18 |

**Figure S3.** All output results for design example 2, which have the AVG WFE RMS smaller than 0.075 *λ*.


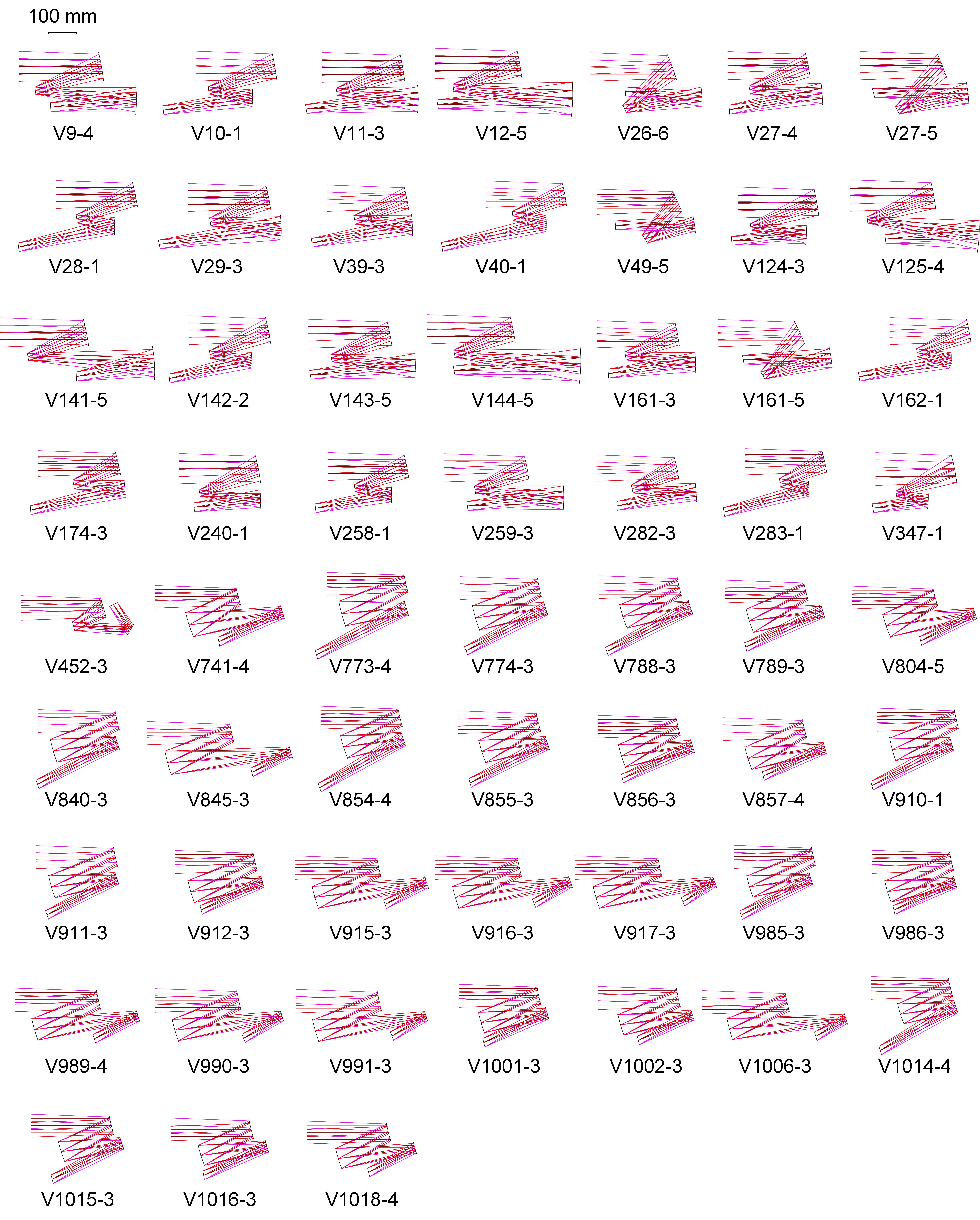


**Table S3.** Minimum, maximum, average, and standard deviation RMS values of the wavefront error across the field-of-view and the distortion percentage (maximum) of all output results of design example 2.

| System number: V#M-#N | | Minimum | Maximum | Average | Standard Deviation | Distortion Percentage |
| --- | --- | --- | --- | --- | --- | --- |
| M | N |  |  |  |  |  |
| 9 | 4 | 0.0274 | 0.0681 | 0.0418 | 0.0106 | 0.09 |
| 10 | 1 | 0.0100 | 0.0486 | 0.0290 | 0.0099 | 0.13 |
| 11 | 3 | 0.0155 | 0.0689 | 0.0390 | 0.0129 | 0.09 |
| 12 | 5 | 0.0238 | 0.1041 | 0.0625 | 0.0174 | 0.09 |
| 26 | 6 | 0.0242 | 0.1122 | 0.0715 | 0.0235 | 0.50 |
| 27 | 4 | 0.0135 | 0.0719 | 0.0402 | 0.0125 | 0.12 |
| 27 | 5 | 0.0209 | 0.0565 | 0.0383 | 0.0100 | 0.56 |
| 28 | 1 | 0.0066 | 0.0441 | 0.0244 | 0.0099 | 0.18 |
| 29 | 3 | 0.0193 | 0.0927 | 0.0496 | 0.0171 | 0.14 |
| 39 | 3 | 0.0079 | 0.0789 | 0.0437 | 0.0146 | 0.12 |
| 40 | 1 | 0.0048 | 0.0816 | 0.0472 | 0.0191 | 0.29 |
| 49 | 5 | 0.0311 | 0.1059 | 0.0707 | 0.0165 | 0.72 |
| 124 | 3 | 0.0107 | 0.0483 | 0.0268 | 0.0098 | 0.02 |
| 125 | 4 | 0.0209 | 0.0791 | 0.0486 | 0.0138 | 0.25 |
| 141 | 5 | 0.0244 | 0.1184 | 0.0647 | 0.0258 | 0.31 |
| 142 | 2 | 0.0290 | 0.0749 | 0.0479 | 0.0124 | 0.15 |
| 143 | 5 | 0.0152 | 0.0459 | 0.0253 | 0.0068 | 0.06 |
| 144 | 5 | 0.0170 | 0.0896 | 0.0540 | 0.0155 | 0.09 |
| 161 | 3 | 0.0137 | 0.1003 | 0.0575 | 0.0195 | 0.07 |
| 161 | 5 | 0.0252 | 0.0718 | 0.0448 | 0.0122 | 0.57 |
| 162 | 1 | 0.0115 | 0.0953 | 0.0456 | 0.0219 | 0.13 |
| 174 | 3 | 0.0199 | 0.0959 | 0.0552 | 0.0162 | 0.13 |
| 240 | 1 | 0.0193 | 0.0551 | 0.0341 | 0.0090 | 0.06 |
| 258 | 1 | 0.0094 | 0.0657 | 0.0368 | 0.0125 | 0.09 |
| 259 | 3 | 0.0110 | 0.0723 | 0.0453 | 0.0148 | 0.03 |
| 282 | 3 | 0.0191 | 0.1174 | 0.0691 | 0.0235 | 0.05 |
| 283 | 1 | 0.0154 | 0.0998 | 0.0493 | 0.0191 | 0.23 |
| 347 | 1 | 0.0223 | 0.0892 | 0.0554 | 0.0158 | 0.16 |
| 452 | 3 | 0.0374 | 0.1272 | 0.0746 | 0.0245 | 0.77 |
| 741 | 4 | 0.0338 | 0.1284 | 0.0724 | 0.0235 | 0.65 |
| 773 | 4 | 0.0108 | 0.1401 | 0.0664 | 0.0365 | 0.31 |
| 774 | 3 | 0.0206 | 0.1595 | 0.0694 | 0.0357 | 0.10 |
| 788 | 3 | 0.0220 | 0.1121 | 0.0620 | 0.0230 | 0.25 |
| 789 | 3 | 0.0163 | 0.1193 | 0.0606 | 0.0270 | 0.39 |
| 804 | 5 | 0.0303 | 0.1200 | 0.0663 | 0.0210 | 0.82 |
| 840 | 3 | 0.0076 | 0.1405 | 0.0620 | 0.0329 | 0.14 |
| 845 | 3 | 0.0174 | 0.0882 | 0.0499 | 0.0155 | 0.31 |
| 854 | 4 | 0.0150 | 0.0773 | 0.0464 | 0.0139 | 0.25 |
| 855 | 3 | 0.0151 | 0.1003 | 0.0516 | 0.0239 | 0.37 |
| 856 | 3 | 0.0155 | 0.1062 | 0.0598 | 0.0244 | 0.54 |
| 857 | 4 | 0.0257 | 0.1212 | 0.0653 | 0.0227 | 0.63 |
| 910 | 1 | 0.0158 | 0.1431 | 0.0652 | 0.0333 | 0.43 |
| 911 | 3 | 0.0163 | 0.1262 | 0.0610 | 0.0286 | 0.25 |
| 912 | 3 | 0.0234 | 0.1271 | 0.0740 | 0.0249 | 0.49 |
| 915 | 3 | 0.0269 | 0.0984 | 0.0610 | 0.0188 | 0.23 |
| 916 | 3 | 0.0148 | 0.1069 | 0.0584 | 0.0217 | 0.19 |
| 917 | 3 | 0.0212 | 0.1123 | 0.0623 | 0.0219 | 0.16 |
| 985 | 3 | 0.0115 | 0.0992 | 0.0425 | 0.0232 | 0.31 |
| 986 | 3 | 0.0127 | 0.1250 | 0.0684 | 0.0249 | 0.54 |
| 989 | 4 | 0.0124 | 0.0631 | 0.0376 | 0.0117 | 0.27 |
| 990 | 3 | 0.0152 | 0.0827 | 0.0516 | 0.0165 | 0.18 |
| 991 | 3 | 0.0128 | 0.0968 | 0.0585 | 0.0207 | 0.17 |
| 1001 | 3 | 0.0115 | 0.1296 | 0.0601 | 0.0280 | 0.50 |
| 1002 | 3 | 0.0295 | 0.1233 | 0.0661 | 0.0235 | 0.55 |
| 1006 | 3 | 0.0290 | 0.1212 | 0.0744 | 0.0262 | 0.22 |
| 1014 | 4 | 0.0123 | 0.0897 | 0.0523 | 0.0195 | 0.49 |
| 1015 | 3 | 0.0226 | 0.0765 | 0.0478 | 0.0134 | 0.66 |
| 1016 | 3 | 0.0168 | 0.1020 | 0.0625 | 0.0192 | 0.79 |
| 1018 | 4 | 0.0307 | 0.0944 | 0.0597 | 0.0157 | 0.67 |

**Figure S4**. The proposed method is used to design a system that have the same specifications and similar volume as the design reported in [21]. The system has a square field-of-view of 4°×4°, a focal length of 600 mm and an entrance pupil of 200 mm. A coarse search is conducted. After 22.2 hours of calculation on a working station having 96 cores 192 threads (2.29 GHz), 48 systems are obtained, which have the AVG WFE RMS varying from 0.11*λ* to 0.30*λ*. These systems can be used as the starting point for further fine search.


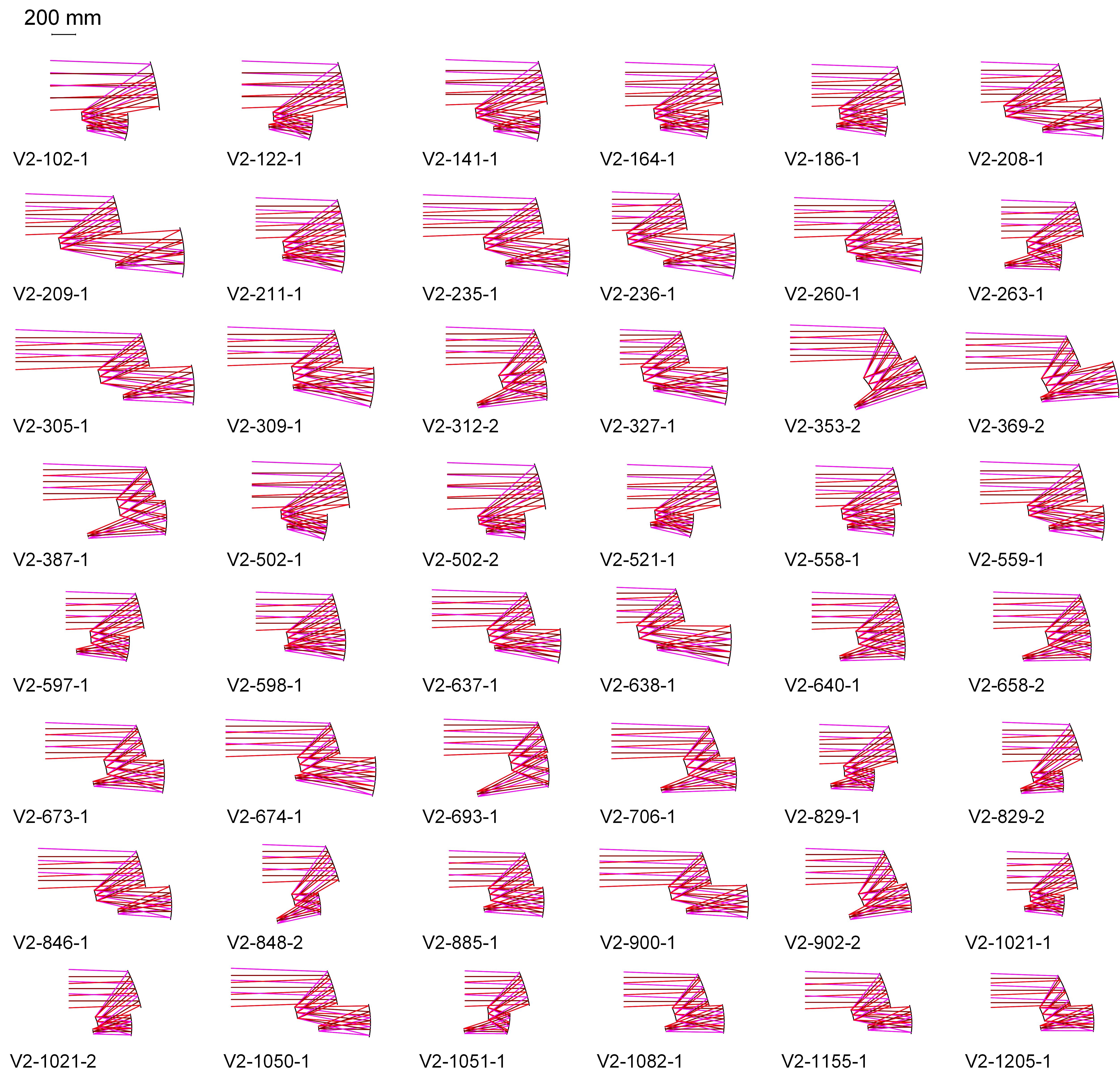


**Table S4.** Minimum, maximum, average, and standard deviation RMS values of the wavefront error across the field-of-view and the distortion percentage (maximum) of the systems in Figure S4.

| System number: V2-#M-#N | | Minimum | Maximum | Average | Standard Deviation | Distortion Percentage |
| --- | --- | --- | --- | --- | --- | --- |
| M | N |  |  |  |  |  |
| 102 | 1 | 0.0742 | 0.3378 | 0.2179 | 0.0702 | 0.62 |
| 122 | 1 | 0.1049 | 0.4002 | 0.2467 | 0.0812 | 0.22 |
| 141 | 1 | 0.0574 | 0.2665 | 0.1565 | 0.0542 | 0.77 |
| 164 | 1 | 0.1742 | 0.5362 | 0.2861 | 0.0939 | 0.29 |
| 186 | 1 | 0.0992 | 0.3484 | 0.2128 | 0.0597 | 0.15 |
| 208 | 1 | 0.0226 | 0.3114 | 0.1519 | 0.0731 | 0.74 |
| 209 | 1 | 0.0955 | 0.2926 | 0.1958 | 0.0424 | 0.92 |
| 211 | 1 | 0.0607 | 0.5275 | 0.2724 | 0.1040 | 0.09 |
| 235 | 1 | 0.1144 | 0.3569 | 0.2628 | 0.0670 | 0.51 |
| 236 | 1 | 0.0636 | 0.4982 | 0.2936 | 0.1029 | 0.51 |
| 260 | 1 | 0.0818 | 0.4316 | 0.2946 | 0.0897 | 0.12 |
| 263 | 1 | 0.1062 | 0.3072 | 0.2038 | 0.0569 | 0.57 |
| 305 | 1 | 0.0746 | 0.4986 | 0.2496 | 0.0797 | 0.34 |
| 309 | 1 | 0.0743 | 0.3958 | 0.2262 | 0.0805 | 0.14 |
| 312 | 2 | 0.1167 | 0.3878 | 0.2653 | 0.0718 | 0.23 |
| 327 | 1 | 0.1781 | 0.5634 | 0.2722 | 0.0869 | 0.10 |
| 353 | 2 | 0.0508 | 0.4637 | 0.2949 | 0.1068 | 0.30 |
| 369 | 2 | 0.0995 | 0.3901 | 0.2086 | 0.0558 | 0.20 |
| 387 | 1 | 0.1744 | 0.3871 | 0.2427 | 0.0469 | 0.80 |
| 502 | 1 | 0.0801 | 0.3225 | 0.2064 | 0.0652 | 0.61 |
| 502 | 2 | 0.0447 | 0.4397 | 0.2914 | 0.0843 | 0.51 |
| 521 | 1 | 0.0501 | 0.3475 | 0.1670 | 0.0660 | 0.19 |
| 558 | 1 | 0.0841 | 0.4099 | 0.2299 | 0.0703 | 0.20 |
| 559 | 1 | 0.0681 | 0.3939 | 0.2178 | 0.0736 | 0.56 |
| 597 | 1 | 0.0956 | 0.2970 | 0.1900 | 0.0499 | 0.21 |
| 598 | 1 | 0.0563 | 0.2277 | 0.1394 | 0.0451 | 0.05 |
| 637 | 1 | 0.1379 | 0.3444 | 0.2126 | 0.0541 | 0.10 |
| 638 | 1 | 0.0369 | 0.3150 | 0.1931 | 0.0731 | 0.21 |
| 640 | 1 | 0.0506 | 0.2277 | 0.1435 | 0.0439 | 0.41 |
| 658 | 2 | 0.0923 | 0.3484 | 0.2175 | 0.0625 | 0.36 |
| 673 | 1 | 0.1283 | 0.4338 | 0.1975 | 0.0637 | 0.10 |
| 674 | 1 | 0.0656 | 0.4303 | 0.2463 | 0.0848 | 0.16 |
| 693 | 1 | 0.1192 | 0.5750 | 0.2842 | 0.0665 | 1.00 |
| 706 | 1 | 0.0752 | 0.4447 | 0.2463 | 0.1032 | 0.41 |
| 829 | 1 | 0.0696 | 0.2667 | 0.1305 | 0.0394 | 0.16 |
| 829 | 2 | 0.0308 | 0.2262 | 0.1337 | 0.0518 | 0.12 |
| 846 | 1 | 0.0313 | 0.2410 | 0.1658 | 0.0456 | 0.38 |
| 848 | 2 | 0.0590 | 0.3707 | 0.1863 | 0.0642 | 0.23 |
| 885 | 1 | 0.0240 | 0.2264 | 0.1102 | 0.0356 | 0.12 |
| 900 | 1 | 0.0709 | 0.4473 | 0.2553 | 0.0706 | 0.24 |
| 902 | 2 | 0.0386 | 0.1851 | 0.1176 | 0.0448 | 0.27 |
| 1021 | 1 | 0.0879 | 0.4668 | 0.1981 | 0.0770 | 0.19 |
| 1021 | 2 | 0.0921 | 0.2760 | 0.1689 | 0.0475 | 0.38 |
| 1050 | 1 | 0.0774 | 0.3373 | 0.1840 | 0.0623 | 0.29 |
| 1051 | 1 | 0.1861 | 0.3925 | 0.2753 | 0.0576 | 0.45 |
| 1082 | 1 | 0.1343 | 0.5300 | 0.2590 | 0.0937 | 0.08 |
| 1155 | 1 | 0.0487 | 0.4330 | 0.2673 | 0.0947 | 0.29 |
| 1205 | 1 | 0.0858 | 0.5517 | 0.2616 | 0.0866 | 0.13 |

**Figure S5.** The proposed method is used to design a system that have the same specifications and similar volume as the design reported in [21]. The system has a square field-of-view of 4°×4°, a focal length of 600 mm and an entrance pupil of 200 mm. Taking the system V2-885-1 in Figure S4 as the centre, a fine search is conducted to solve for designs that have higher imaging quality, e.g. with the AVG WFE RMS smaller than 0.075*λ*. After 12.7 hours of calculation, 5 systems that have the AVG WFE RMS smaller than 0.11*λ* are obtained and are presented in this figure, among which the AVG WFE RMS of system V2-885-1-34-1 reaches 0.064*λ*. In addition, 31 systems with the AVG WFE RMS varying from 0.11*λ* and 0.2*λ* are obtained.


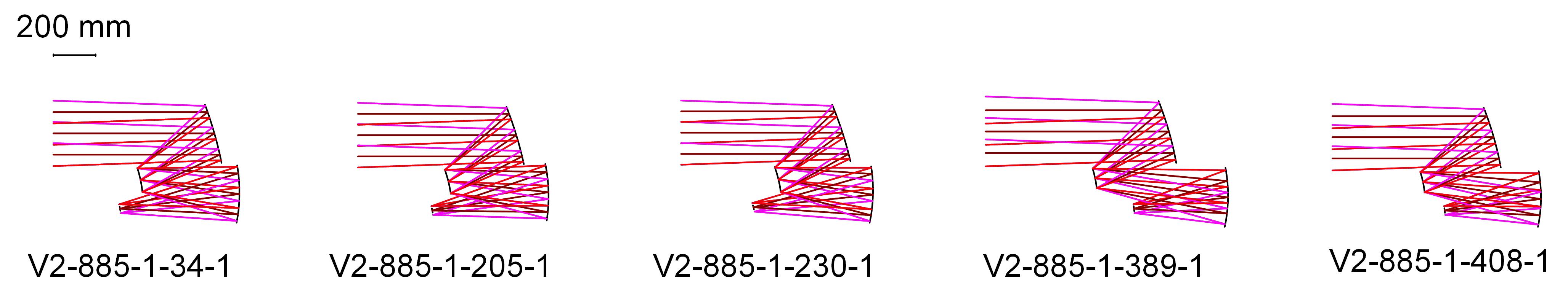


**Table S5.** Minimum, maximum, average, and standard deviation RMS values of the wavefront error across the field-of-view and the distortion percentage (maximum) of the systems in Figure S5.

| System number: V2-885-1-#M-#N | | Minimum | Maximum | Average | Standard Deviation | Distortion Percentage |
| --- | --- | --- | --- | --- | --- | --- |
| M | N |  |  |  |  |  |
| 34 | 1 | 0.0448 | 0.1022 | 0.0638 | 0.0122 | 0.11 |
| 205 | 1 | 0.0334 | 0.1533 | 0.0818 | 0.0257 | 0.07 |
| 230 | 1 | 0.0439 | 0.1184 | 0.0837 | 0.0189 | 0.12 |
| 389 | 1 | 0.0346 | 0.1856 | 0.1034 | 0.0371 | 0.35 |
| 408 | 1 | 0.0299 | 0.1339 | 0.0792 | 0.0270 | 0.20 |

**Table S6.** Inputs and parameters for the design of a system with a focal length of 600 mm and an entrance pupil of 200 mm.

|  |  | Coarse search | Fine search |
| --- | --- | --- | --- |
| Input | Full field angles (°) | ±2 in X, ±2 in Y | |
|  | Entrance pupil diameter (EPD) (mm) | 600 | |
|  | Focal length (mm) | 200 | |
|  | F number | 3 | |
|  | Working band | VIS (420-680 nm) | |
|  | Primary wavelength | 587.6 nm | |
| Phase 1 | Range and interval of the curvature radius for spherical surfaces (mm) | *r*_1_∈[−2000, 0], Δ*r*_1_=200,  *r*_2_∈[−1000, 0], Δ*r*_2_=100,  *r*_3_∈[−1000, 0], Δ*r*_3_=100 | *r*_1_∈[−1800, −1400], Δ*r*_1_=100,  *r*_2_∈[−600, −400], Δ*r*_2_=50,  *r*_3_∈[−800, −600], Δ*r*_3_=50 |
|  | Structure constraints | EPD<\|*d*_1_\|<5×EPD  Δ*d*_1_=EPD/8 | 375 mm<\|*d*_1_\|<425 mm  Δ*d*_1_=12.5 mm |
|  |  | *d*_1_<0, *d*_2_>0, *d*_3_<0,  −2≤*d*_2_/*d*_1_≤−1/2, 1/2≤*d*_3_/*d*_1_≤2 | |
|  | Total number of OP distributions in coaxial systems | 1378 | 521 |
| Phase 2 | Range and interval of the angles in the structure vector | *θ*_1_∈(−180°, −120°],  *θ*_2_∈[120°, 180°],  *θ*_3_∈[−180°, −120°] | |
|  |  | Δ*θ*_1_=2°, Δ*θ*_2,3_=5° | |
|  | Total number of computing jobs | 1995 | 851 |
| Phase 3 | Method to correct the OP of the entire system and construct freeform systems | Point-by-point construction method | |
|  | Field angles sampled over the half field angle | (0, 0) (0, 2) (0, −2)  (2, 0) (2, 2) (2, −2) | |
|  | Number of feature light rays sampled over the full aperture for one field angle | 101 | |
| Phase 4 | Method to improve the imaging quality | Point-by-point iteration method | |
|  | Field angles sampled over the half field angle | (0, 0) (0, 2) (0, −2)  (2, 0) (2, 2) (2, −2) | |
|  | Number of feature light rays sampled over the full aperture for one field angle | 101 | |
|  | Iteration termination condition: imaging quality threshold | *σ*_itr_=0 | |
|  | Iteration termination condition: imaging quality improvement rate threshold | *τ*_itr_=3% | |
|  | Method to solve for the optimal image plane tilt angle | One-dimensional searching | |
|  | Range and interval for the one-dimensional search of image plane tilt angle (°) | *β*∈[*β*_0_−2, *β*_0_+2], Δ*β*=1 | |
|  | One-dimensional searching termination condition: imaging quality threshold | *σ*_srh_=0.001 | |
|  | One-dimensional searching termination condition: imaging quality improvement rate threshold | *τ*_srh_=0.3% | |
| Phase 5 | Imaging quality requirement | AVG RMS WFE < 0.3*λ* | AVG RMS WFE < 0.075*λ* |

**Table S7.** Inputs and parameters for the proposed automatic design paradigm used for design example 1 and 2.

|  |  | Design example 1 | The localized design | Design example 2 |
| --- | --- | --- | --- | --- |
| Input | Full field angles (°) | ±4 in X, ±3 in Y | | ±2 in X, ±2 in Y |
|  | Entrance pupil diameter (EPD) (mm) | 27.78 | | 50 |
|  | Focal length (mm) | 50 | | 450 |
|  | F number | 1.8 | | 9 |
|  | Working band | LWIR (8-14 μm) | | VIS (420-680 nm) |
|  | Primary wavelength | 10 μm | | 587.6 nm |
| Phase 1 | Range and interval of the curvature radius for spherical surfaces (mm) | *r*_1,2,3_∈[−1000, 1000],  Δ*r*_1,2,3_=100 | *r*_1_∈[−1000, −700], Δ*r*_1_=150,  *r*_2_∈[−300, −200], Δ*r*_2_=25,  *r*_3_∈[−120, −80], Δ*r*_3_=20 | *r*_1,2,3_∈[−1000, 1000],  Δ*r*_1,2,3_=100 |
|  | Structure constraints | EPD<\|*d*_1_\|<4×EPD  Δ*d*_1_=EPD/2 | 40 mm<\|*d*_1_\|<56 mm  Δ*d*_1_=14 mm | EPD/2<\|*d*_1_\|<5×EPD  Δ*d*_1_=EPD/2 |
|  |  | *d*_1_<0, *d*_2_>0, *d*_3_<0,  −2≤*d*_2_/*d*_1_≤−1/2, 1/2≤*d*_3_/*d*_1_≤2, *d*_3_≤−2×EPD | | |
|  | Total number of OP distributions in coaxial systems | 377 | 73 | 1053 |
| Phase 2 | Range and interval of the angles in the structure vector | *θ*_1_∈(−180°, −120°],  *θ*_2_∈[120°, 240°],  *θ*_3_∈[−240°, −120°] | | |
|  |  | Δ*θ*_1,2,3_=5° | | Δ*θ*_1,2,3_=20° |
|  | Total number of computing jobs | 1178 | 217 | 3196 |
| Phase 3 | Method to correct the OP of the entire system and construct freeform systems | Point-by-point construction method | | |
|  | Field angles sampled over the half field angle | (0, 0) (0, 3) (0, −3)  (4, 0) (4, 3) (4, −3) | | (0, 0) (0, 2) (0, −2)  (2, 0) (2, 2) (2, −2) |
|  | Number of feature light rays sampled over the full aperture for one field angle | 97 | | |
| Phase 4 | Method to improve the imaging quality | Point-by-point iteration method | | |
|  | Field angles sampled over the half field angle | (0, 0) (0, 3) (0, −3)  (4, 0) (4, 3) (4, −3) | | (0, 0) (0, 2) (0, −2)  (2, 0) (2, 2) (2, −2) |
|  | Number of feature light rays sampled over the full aperture for one field angle | 97 | | |
|  | Iteration termination condition: imaging quality threshold | *σ*_itr_=0.003 | | |
|  | Iteration termination condition: imaging quality improvement rate threshold | *τ*_itr_=3% | | |
|  | Method to solve for the optimal image plane tilt angle | One-dimensional searching | | |
|  | Range and interval for the one-dimensional search of image plane tilt angle (°) | *β*∈[*β*_0_−5, *β*_0_+5], Δ*β*=1 | | |
|  | One-dimensional searching termination condition: imaging quality threshold | *σ*_srh_=0.0059 | | *σ*_srh_=0.005 |
|  | One-dimensional searching termination condition: imaging quality improvement rate threshold | *τ*_srh_=0.5% | | |
| Phase 5 | Imaging quality requirement | AVG RMS WFE < 0.075*λ* | | |

**Figure S6.** Framework for the automatic design method for three-mirror freeform systems.


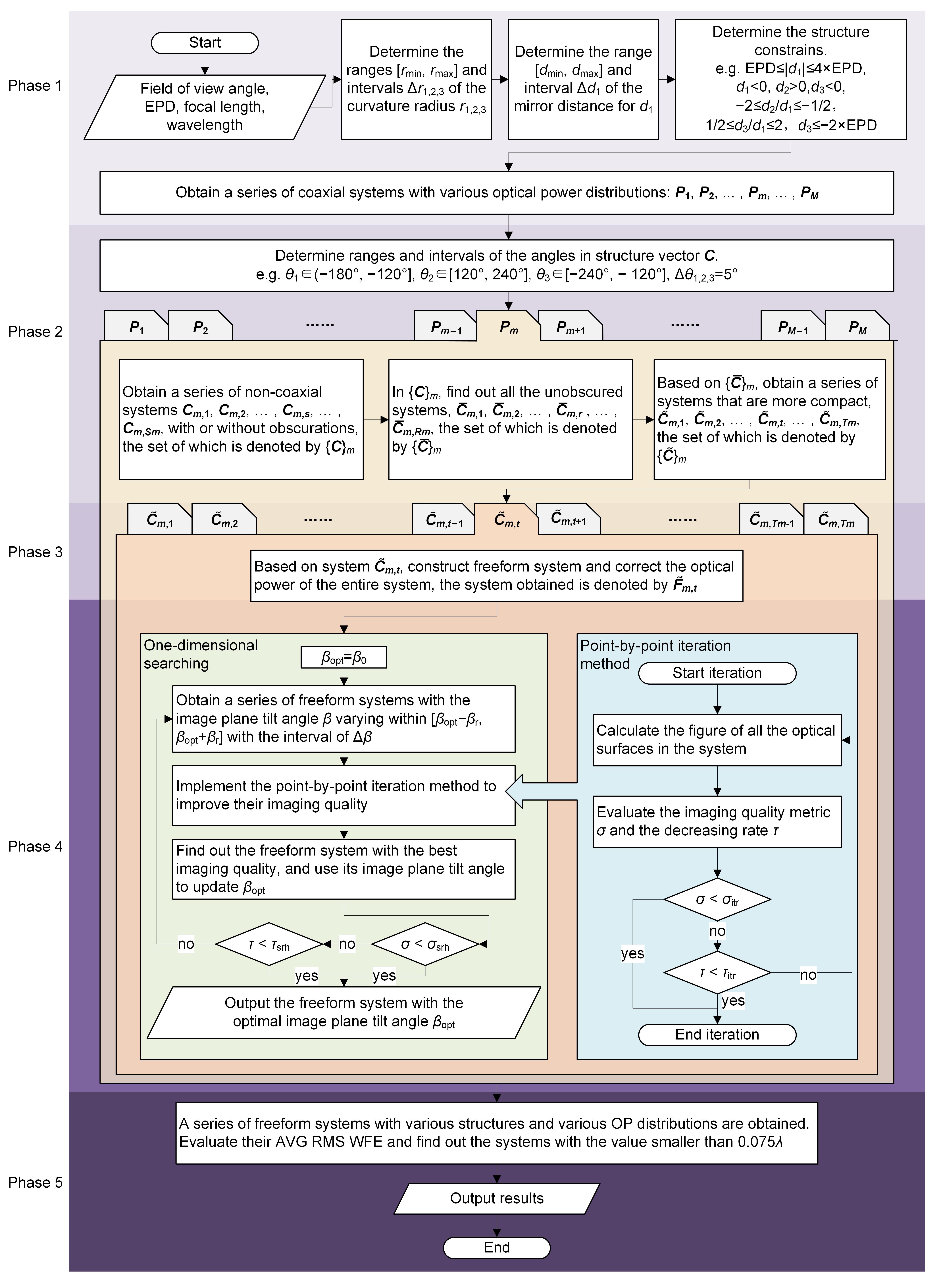

Supplement: Supplementary file 1 — Supplementary Information for Towards automatic freeform optics design: coarse and fine search of the three-mirror solution space [file 41377_2021_510_MOESM1_ESM.docx]
